# Supplementary material for: Adjusting for genetic confounders in transcriptome-wide association studies improves discovery of risk genes of complex traits
Source: Nat Genet. 2024 Jan 26;56(2):336–47. doi: 10.1038/s41588-023-01648-9 (PMC10864181; doi:10.1038/s41588-023-01648-9)
Supplement: Supplementary file 1 — Supplementary Note and Supplementary Figs. 1–19. [file 41588_2023_1648_MOESM1_ESM.pdf]

nature genetics

Technical Report https://doi.org/10.1038/s41588-023-01648-9

Adjusting for genetic confounders in transcriptome-wide association studies improves discovery of risk genes of complex traits

In the format provided by the authors and unedited

Supplementary Notes

EM algorithm for estimating the prior parameters

We first restate our full model as described in Methods. We have a linear model with K groups of explanatory variables:

$$y = \sum_{k=1}^{K} \sum_{j \in M_k} \beta_j X_j + \epsilon, \qquad \epsilon \sim N(0, \sigma^2), \tag{1}$$

where $X_j, 1 \leq j \leq p$, is j-th explanatory variable and $j \in M_k$ denotes that it belongs to the group k. The effect size β_j's follow spike-and-slab prior distributions with group-specific parameters. Let γ_j be an indicator of whether X_j has non-zero effect:

$$\begin{array}{ll} \gamma_j & \sim \text{Bernoulli}(\pi_k) \\ \beta_j | \gamma_j = 1 & \sim N(0, \sigma_k^2) \\ \beta_j | \gamma_j = 0 & \sim \delta_0. \end{array} \tag{2}$$

For simplicity, we assume σ^2 is given (see Methods). Our goal is to estimate the prior parameters $\theta = \{\pi_k, \sigma_k^2, 1 \leq k \leq K\}$.

We use Expectation-Maximization (EM) algorithm to estimate these parameters, treating the effect sizes $\boldsymbol{\beta} = (\beta_1, \cdots, \beta_p)$ and the configuration of all indicators $\boldsymbol{\Gamma} = (\gamma_1, \cdots, \gamma_p)$ as missing data. The complete data log-likelihood function is given by:

$$\log P(\boldsymbol{y}, \boldsymbol{\Gamma}, \boldsymbol{\beta} | \boldsymbol{X}, \theta) = \log P(\boldsymbol{\Gamma}|\theta) + \log P(\boldsymbol{y}, \boldsymbol{\beta} | \boldsymbol{X}, \boldsymbol{\Gamma}, \theta) \tag{3}$$

$$= \log P(\boldsymbol{\Gamma}|\theta) + \log P(\boldsymbol{\beta}|\boldsymbol{\Gamma}, \theta) + \log P(\boldsymbol{y}|\boldsymbol{X}, \boldsymbol{\beta}) \tag{4}$$

$$= \sum_{k=1}^{K} \sum_{j \in M_k} \log P(\gamma_j | \pi_k) + \sum_{k=1}^{K} \sum_{j \in M_k} \log P(\beta_j | \gamma_j, \sigma_k^2) + \log P(\boldsymbol{y}|\boldsymbol{X}, \boldsymbol{\beta}). \tag{5}$$

Note that the last term does not depend on the prior parameters θ, so we can ignore it in the EM algorithm.

In the E-step, we take expectation of the complete log-likelihood function over $\boldsymbol{\beta}, \boldsymbol{\Gamma} | \theta^{(t)}$, where $\theta^{(t)}$ is the parameters from step t:

$$Q(\theta|\theta^{(t)}) = \mathbb{E}_{\boldsymbol{\beta}, \boldsymbol{\Gamma}} \log P(\boldsymbol{y}, \boldsymbol{\Gamma}, \boldsymbol{\beta} | \boldsymbol{X}, \theta, \sigma) \tag{6}$$

$$= \sum_{k=1}^{K} \sum_{j \in M_k} \mathbb{E}_{\gamma_j} \log P(\gamma_j | \pi_k) + \sum_{k=1}^{K} \sum_{j \in M_k} \mathbb{E}_{\beta_j, \gamma_j} \log P(\beta_j | \gamma_j, \sigma_k^2) + \text{constant}. \tag{7}$$

We can simplify this function. First, we note:

$$\mathbb{E}_{\gamma_j} \log P(\gamma_j|\pi_k) = \mathbb{E}_{\gamma_j} [\gamma_j \log \pi_k + (1 - \gamma_j) \log(1 - \pi_k)] = \alpha_j^{(t)} \log \pi_k + (1 - \alpha_j^{(t)}) \log(1 - \pi_k), \tag{8}$$

where $\alpha_j^{(t)} = P(\gamma_j = 1 | \theta^{(t)}, \boldsymbol{X}, \boldsymbol{y})$ is the posterior inclusion probability of variable j at the t-th iteration. Next,

$$\mathbb{E}_{\beta_j, \gamma_j} \log P(\beta_j | \gamma_j, \sigma_k^2)) = \mathbb{E}_{\beta_j, \gamma_j} [\gamma_j \log P(\beta_j | \gamma_j = 1, \sigma_k^2) + (1 - \gamma_j) \log P(\beta_j | \gamma_j = 0)] \tag{9}$$

$$= \alpha_j^{(t)} \cdot \mathbb{E}_{\beta_j|\gamma_j=1} [\log P(\beta_j | \gamma_j = 1, \sigma_k^2)] + (1 - \alpha_j^{(t)}) \cdot \mathbb{E}_{\beta_j|\gamma_j=0} [\log P(\beta_j | \gamma_j = 0)]. \tag{10}$$

Given that $\beta_j = 0$ when $\gamma_j = 0$, the second term in the expectation is simply 0. For the first term, we plug

in the normal density $\beta_j \sim N(0, \sigma_k^2)$:

$$\mathbb{E}_{\beta_j,\gamma_j} \log P(\beta_j|\gamma_j, \sigma_k^2)) = \alpha_j^{(t)} \cdot \mathbb{E}_{\beta_j|\gamma_j=1} \left(-\log\sqrt{2\pi}\sigma_k - \frac{\beta_j^2}{2\sigma_k^2} \right) = \alpha_j^{(t)} \cdot \left(-\log\sqrt{2\pi}\sigma_k - \frac{\tau_j^{2,(t)}}{2\sigma_k^2} \right), \tag{11}$$

where $\tau_j^{2,(t)} = \mathbb{E}(\beta_j^2|\gamma_j = 1, \boldsymbol{X}, \boldsymbol{y}, \theta^{(t)})$ is the second moment of posterior effect size distribution of β_j given the current parameter estimate θ^t and that $\gamma_j = 1$.

Putting all these together, we have:

$$Q(\theta|\theta^{(t)}) = \sum_{k=1}^{K} \sum_{j\in M_k} \left[\alpha_j^{(t)} \log \pi_k + (1-\alpha_j^{(t)}) \log(1-\pi_k) \right] + \sum_{k=1}^{K} \sum_{j\in M_k} \alpha_j^{(t)} \left(-\log\sqrt{2\pi}\sigma_k - \frac{\tau_j^{2,(t)}}{2\sigma_k^2} \right) \tag{12}$$

At the M-step, we find θ that maximize $Q(\theta|\theta^t)$. We begin with the update rule for π_k, noting that only the first term contains π_k.

$$\frac{\partial Q(\theta|\theta^{(t)})}{\partial \pi_k} = \sum_{j\in M_k} \left[\frac{\alpha_j^{(t)}}{\pi_k} - \frac{1-\alpha_j^{(t)}}{1-\pi_k} \right] = 0. \tag{13}$$

Solving this equation gives the update rule of π_k:

$$\pi_k^{(t+1)} = \frac{1}{|M_k|} \sum_{j\in M_k} \alpha_j^{(t)}, \tag{14}$$

where $|M_k|$ is the size of M_k. Next we derive the update rule of σ_k^2:

$$\frac{\partial Q(\theta|\theta^{(t)})}{\partial \sigma_k} = \sum_{j\in M_k} \alpha_j^{(t)} \left[-\frac{1}{\sigma_k} + \frac{\tau_j^{2,(t)}}{\sigma_k^3} \right] = 0. \tag{15}$$

This leads to the update rule for σ_k^2 as:

$$\sigma_k^{2,(t+1)} = \frac{\sum_{j\in M_k} \alpha_j^{(t)} \tau_j^{2,(t)}}{\sum_{j\in M_k} \alpha_j^{(t)}}. \tag{16}$$

The computation of $\alpha_j^{(t)}$ and $\tau_j^{2,(t)}$ at each iteration will be described below. We perform E and M step until each prior parameters change < 0.00001 in one iteration or the maximum number of iterations is met.

Calculation of α_j and τ_j^2 under the single effect approximation.

In the EM iteration, we need to compute the PIP $\alpha_j^{(t)}$ and the posterior second moment $\tau_j^{2,(t)}$ of each variable (the index (t) will be dropped later for simplicity). To simplify, we analyze each block one at a time, assuming each block has at most a single variable with non-zero effects. We further assume that each single block explains a minimal variance of $\boldsymbol{y}$, so we set $\sigma = 1$ in the calculation. Let Γ_j be the configuration where $\gamma_j = 1$ and all other indicators are 0, and $\boldsymbol{\Gamma}_0$ be the null configuration where $\gamma_j = 0$ for all variables. Let p_j be the prior inclusion probability of variable j, which depends on the group variable j belongs to, $p_j = \pi_k$ when $j \in M_k$. The prior probabilities of the configurations are given by:

$$P(\boldsymbol{\Gamma}_0|\theta) = \prod_j (1 - p_j), \tag{17}$$

$$P(\mathbf{\Gamma}_j|\theta) = \frac{p_j}{1-p_j} \cdot P(\mathbf{\Gamma}_0|\theta). \tag{18}$$

For variable j, we define its Bayes factor, as the probability of data under $\mathbf{\Gamma}_j$ vs. under $\mathbf{\Gamma}_0$. Based on the Wakefield formula[1], it is given by:

$$\begin{aligned} B_j &:= \frac{P(\boldsymbol{y}|\boldsymbol{X}, \mathbf{\Gamma}_j, \theta)}{P(\boldsymbol{y}|\boldsymbol{X}, \mathbf{\Gamma}_0, \theta)} & (19) \\ &= \sqrt{\frac{s_j^2}{\sigma_k^2 + s_j^2}} \cdot \exp\left(\frac{\hat{\beta_j}^2 \sigma_k^2}{2s_j^2(\sigma_k^2 + s_j^2)}\right), & (20) \end{aligned}$$

where σ_k is the prior variance of effect size for variables in group k. $\hat{\beta}_j$ is the estimated effect size of variable j, and s_j is the standard error. They are given by:

$$\begin{aligned} \hat{\beta}_j &:= \boldsymbol{X}_j^T \boldsymbol{y}/n, & (21) \\ s_j^2 &:= (\boldsymbol{y} - \boldsymbol{X}_j\hat{\beta}_j)^T(\boldsymbol{y} - \boldsymbol{X}_j\hat{\beta}_j)/(n\boldsymbol{X}_j^T\boldsymbol{X}_j) \approx 1/n. & (22) \end{aligned}$$

The posterior inclusion probabilities depend on the prior and the BFs of variables:

$$\begin{aligned} \alpha_j &= P(\gamma_j = 1|\boldsymbol{X}, \boldsymbol{y}, \theta) & (23) \\ &= P(\mathbf{\Gamma}_j|\boldsymbol{X}, \boldsymbol{y}, \theta) & (24) \\ &= \frac{P(\boldsymbol{y}|\boldsymbol{X}, \mathbf{\Gamma}_j, \theta)P(\mathbf{\Gamma}_j|\theta)}{P(\boldsymbol{y}|\boldsymbol{X}, \mathbf{\Gamma}_0, \theta)P(\mathbf{\Gamma}_0|\theta) + \sum_j P(\boldsymbol{y}|\boldsymbol{X}, \gamma_j, \theta)P(\mathbf{\Gamma}_j|\theta)} & (25) \\ &= \frac{P(\mathbf{\Gamma}_j|\theta) \cdot B_j}{P(\mathbf{\Gamma}_0|\theta) + \sum_j P(\mathbf{\Gamma}_j|\theta) \cdot B_j} & (26) \\ &= \frac{p_j/(1-p_j)B_j}{1 + \sum_j p_j/(1-p_j)B_j}. & (27) \end{aligned}$$

Following the derivation in the SuSiE paper, the posterior distribution of $\beta_j|\gamma_j = 1$ follows $N(\mu_{1j}, \sigma_{1j}^2)$ where $\sigma_{1j}^2 = s_j^2\sigma_k^2/(\sigma_k^2 + s_j^2)$ and $\mu_{1j} = \sigma_{1j}^2\hat{\beta}_j/s_j^2$, so we have the second moment of posterior distribution,

$$\tau_j^2 = \mathbb{E}(\beta_j^2|\gamma_j = 1, \theta) = \text{var}(\beta_j|\boldsymbol{X}, \boldsymbol{y}, \theta, \gamma_j = 1) + (\mathbb{E}(\beta_j|\boldsymbol{X}, \boldsymbol{y}, \theta, \gamma_j = 1))^2 = \mu_{1j}^2 + \sigma_{1j}^2. \tag{28}$$

When using summary statistics in the form of Z-scores, we replace $\hat{\beta}_j$ as $\hat{z_j}/n$, where $\hat{z_j}$ is the Z-score of variable j. α_j can still be derived by Equation 27 where B_j is given by

$$B_j = \sqrt{\frac{s_j^2}{\sigma_k^2 + s_j^2}} \cdot \exp\left(\frac{\hat{z_j}^2 \sigma_k^2}{2s_j^2(\sigma_k^2 + s_j^2)}\right). \tag{29}$$

where σ_k is the variance of Z score for variables in group k. τ_j^2 can still be derived by Equation 28 where $\mu_{1j} = \sigma_{1j}^2\hat{z}_j$.

We provided some justifications of the single effect assumption. In statistical fine-mapping, several methods have been developed to incorporate functional information of variants to set the prior inclusion probabilities. In such models, it has been found that for the purpose of estimating parameters of the prior distributions, the single effect approximation is often sufficient [2]. Furthermore, in our implementation, we prune large blocks that are likely to contain multiple causal variables in the parameter estimation step. Finally, our simulations showed that the parameters estimated under single-effect assumption were generally

accurate (Figure 2 of the main text).

In the implementation of our method, we used the algorithm of single effect model (SER) from SuSiE to get α_j and τ_j^2. When $p_j \ll 1$, PIP and the second moment of posterior distribution under SuSiE SER should be similar or the same as those from our model. In more detail, we use p_j as prior probabilities in SuSiE and $\pi_0 = 1 - \sum_j p_j$ as null weight, PIP under SuSiE SER α'_j is given by

$$\alpha'_j = \frac{p_j B_j}{\pi_0 + \sum_j p_j B_j} = \frac{p_j/\pi_0 \cdot B_j}{1 + \sum_j p_j/\pi_0 \cdot B_j} \approx \alpha_j. \tag{30}$$

For the approximation, we used $\pi_0 = 1 - \sum_j p_j \approx 1 - p_j$ under the assumption that $p_j \ll 1$. The posterior distribution of $\beta_j|\gamma_j = 1$ under SuSiE SER is the same as in our model.

Similarly, we used the algorithm of the SER model in SuSiE RSS to get α_j and τ_j^2 when the input is summary statistics($\hat{\boldsymbol{z}}, \boldsymbol{R}$).

Initialization and selection of single effect blocks

To have a good chance of working with blocks satisfying the single effect assumption, we compute the prior probability of a block having at most one causal variable, denoted as $p_{\text{single effect}}$. It is given by:

$$p_{\text{single effect}} = P(\boldsymbol{\Gamma}_0) + \sum_j P(\gamma_j) = \prod_j (1 - p_j) \left(1 + \sum_j \frac{p_j}{1 - p_j}\right). \tag{31}$$

Note that this probability considers both cases where a block has no causal variable, and just one. We choose blocks with $p_{\text{single effect}}$ above a threshold, 0.8 in our simulation and real data analysis, to be used in prior parameter estimation. The effect of this block selection step is that large blocks that are likely to contain more than one signal *a priori*, are filtered.

To initialize the EM algorithm described in "Inference of the individual level model" and "Inference of the summary statistics model", we use the same prior parameters for genes and variants. The thinning procedure is applied at the beginning of the EM algorithm if specified. We then run 3 iterations of the EM algorithm using all LD blocks, using this as an initial estimate of p_j. Then, we select single effect blocks based on $p_{\text{single effect}}$ given by Equation (31) and used these for 30 more iterations of the EM algorithm.

Computation of marginal associations $\hat{\boldsymbol{z}}$ and correlation matrix $\boldsymbol{R}$

. When variable j is a gene, we can compute its Z-score, $\hat{z}_j$ following the S-PrediXcan formula [3]. Specifically, suppose the gene j has m SNPs with nonzero weights in its expression prediction model. Let $\boldsymbol{w}_j = (w_{j1}, w_{j2}, .., w_{jm})$ be their standardized weights, $\hat{\boldsymbol{z}}_j^s = (\hat{z}_{j1}, \hat{z}_{j2} ..., \hat{z}_{jm})^T$ be the Z scores of the associations of the variants to the phenotype, and $\boldsymbol{R}_j^s$ be the $m \times m$ correlation matrix, i.e. LD matrix, of these variants. Then $\hat{z}_j$ is basically the weighted sum of the Z-scores of all the m variants:

$$\hat{z}_j = \frac{\boldsymbol{w}_j \hat{\boldsymbol{z}}_j^s}{\sqrt{\boldsymbol{w}_j \boldsymbol{R}_j^s \boldsymbol{w}_j^T}}. \tag{32}$$

We also need $\boldsymbol{R}$, the correlation matrix, of all variables. We denote its element at row i and column j as $R_{ij} = \text{Cor}(\boldsymbol{X}_i, \boldsymbol{X}_j)$, where $\boldsymbol{X}_i$ and $\boldsymbol{X}_j$ are the n-dim. vectors (n is sample size) of the variables i and j. When both variable i and j are variants, R_{ij} is given by the variant LD matrix. When one of the variable, for example $\boldsymbol{X}_j$, is an imputed gene, we can write it as: $\boldsymbol{X}_j = \boldsymbol{X}_j^s \boldsymbol{w}_j^T$, where $\boldsymbol{X}_j^s$ is a $n \times m$ matrix of

genotypes for the m variants in gene j's prediction model. R_{ij} is then given by:

$$R_{ij} = \frac{\text{Cov}\left(\boldsymbol{X}_i, \boldsymbol{X}_j\right)}{\sqrt{\text{Var}(\boldsymbol{X}_i)\text{Var}(\boldsymbol{X}_j)}} = \frac{\text{Cov}\left(\boldsymbol{X}_i, \boldsymbol{X}_j^s \boldsymbol{w}_j^T\right)}{\sqrt{\text{Var}(\boldsymbol{X}_i)\text{Var}(\boldsymbol{X}_j^s \boldsymbol{w}_j^T)}} = \frac{\boldsymbol{R}_{i,j}^s \boldsymbol{w}_j^T}{\sqrt{\boldsymbol{w}_j \boldsymbol{R}_j^s \boldsymbol{w}_j^T}}, \tag{33}$$

where $\boldsymbol{R}_{i,j}^s$ is the m-dimensional vector of correlation between variant i with each of the m variants in the prediction model of gene j. When both variables i and j are genes,

$$R_{ij} = \frac{\text{Cov}\left(\boldsymbol{X}_i, \boldsymbol{X}_j\right)}{\sqrt{\text{Var}(\boldsymbol{X}_i)\text{Var}(\boldsymbol{X}_j)}} = \frac{\text{Cov}\left(\boldsymbol{X}_i^s \boldsymbol{w}_i^T, \boldsymbol{X}_j^s \boldsymbol{w}_j^T\right)}{\sqrt{\text{Var}(\boldsymbol{X}_i^s \boldsymbol{w}_i^T)\text{Var}(\boldsymbol{X}_j^s \boldsymbol{w}_j^T)}} = \frac{\boldsymbol{w}_i \boldsymbol{R}_{i,j}^s \boldsymbol{w}_j^T}{\sqrt{\boldsymbol{w}_i \boldsymbol{R}_i^s \boldsymbol{w}_i^T}\sqrt{\boldsymbol{w}_j \boldsymbol{R}_j^s \boldsymbol{w}_j^T}}, \tag{34}$$

where $\boldsymbol{X}_i^s$ is the genotype matrix and $\boldsymbol{w}_i$ is the vector of standardized weights for variants in gene i's prediction model. $\boldsymbol{R}_i^s$ is the correlation matrix of $\boldsymbol{X}_i^s$. $\boldsymbol{R}_{i,j}^s$ is now the correlation matrix between the variants in the prediction models of gene i and gene j.

Speeding up computation

Parameter estimation is the most time-consuming step. This is especially the case when variants are densely genotyped. We developed a "thinning" procedure to speed up this step. Specifically, we randomly select a fraction of SNPs in the single effect blocks, and only use these variants in the EM update. When most selected blocks have < 1000 variants, the EM algorithm can be done in one or two hours. We have found in our simulations that this thinning step does not affect the accuracy of results. In the final step of analyzing individual blocks, the estimated prior probability for variants from the thinning procedure will be scaled back for the original variants data. This is done by dividing the prior probability by the fraction of selected variants. The other parameters are not affected by this procedure. To reduce computational burden of the final step, we can analyze an individual block with the original variants only when results using the thinned variants showed strong signals for gene effect, e.g the maximum PIP of genes in the block > 0.8. Our software allows the user to specify the desired thinning parameter (the fraction of variants selected) in the parameter estimation step and the PIP threshold used to determine which blocks to analyze with original variants in the final step.

Connection with colocalization methods

We show that under certain conditions, cTWAS reduces to colocalization analysis. Specifically, we assume there is a single causal gene in a region being studied, and that gene has a single causal eQTL variant (known as eQTN in literature). Let X be the expression of the causal gene, $\tilde{X}$ be its *cis*-genetic component, and G_j be the genotype of the eQTN. We have:

$$\tilde{X} = G_j w_j, \tag{35}$$

where w_j is the weight of G_j in the prediction model of X. We also assume that G_j acts on the phenotype only through the gene X, thus its direct effect $\theta_j = 0$. Our model of the phenotype y is given by:

$$y = \tilde{X}\beta + \sum_{i \neq j} G_i \theta_i + \epsilon, \tag{36}$$

where β, θ_i are the causal effects of the gene and variants, respectively. Also note that both β and θ_i's follow spike-and-slab prior with prior inclusion probabilities π_G (gene effect) and π_V (variant effect), respectively.

We can now plug in $\tilde{X} = G_j w_j$ into the model of phenotype:

$$y = G_j w_j \beta + \sum_{i \neq j} G_i \theta_i + \epsilon. \tag{37}$$

We see now that the gene effect $\beta \neq 0$ if and only if the effect of variant j, $w_j \beta \neq 0$. So our problem reduces to a fine-mapping problem, where we determine which variant(s) has non-zero coefficients. The difference with standard fine-mapping is that the prior inclusion probabilities vary with variants. The eQTN variant has prior probability π_G, while other variants have prior π_V, generally smaller than π_G. So the model would effectively perform fine-mapping, favoring the eQTN variant.

The model we described is effectively Enloc, a method for colocalization analysis [4]. We give a short description here of Enloc here. We denote d_j as an indicator of whether G_j is an eQTL, and γ_j as an indicator of whether G_j is a causal variant of the phenotype in GWAS. Colocalization of eQTL and GWAS trait in a region of interest thus means that there is some variant in the region, where $d_j = 1$ and $\gamma_j = 1$. Enloc performs colocalization analysis in several steps. It first performs fine-mapping analysis of eQTL data, estimating $P(d_j = 1)$ for all variants. In the next step, Enloc assesses how often the eQTNs are also causal variants of a complex phenotype from GWAS. This step estimates the conditional distribution $P(\gamma_j | d_j)$. In general, we expect $P(\gamma_j = 1 | d_j = 1) >> P(\gamma_j = 1 | d_j = 0)$. The enrichment of causal variants in eQTNs is reflected by the parameter α_1 in Enloc, defined as the log odds ratio:

$$\alpha_1 = \log \frac{P(\gamma_j = 1 | d_j = 1)/P(\gamma_j = 0 | d_j = 1)}{P(\gamma_j = 1 | d_j = 0)/P(\gamma_j = 0 | d_j = 0)}. \tag{38}$$

Enloc uses the data across genome to estimate α_1. In the final step, Enloc performs fine-mapping of the GWAS trait in any regions of interest, using the prior probabilities of γ_j's estimated in the previous step. Because eQTNs generally have higher prior, this allows Enloc to favor eQTNs in fine-mapping of the trait GWAS. The results of Enloc are expressed as $P(d_j = 1, \gamma_j = 1 | D, \hat{\alpha_1})$, where D means all the data we have.

From this description of Enloc, we can see that the reduced cTWAS model, Equation 37, is almost equivalent to Enloc. Both methods perform fine-mapping of causal variants of a phenotype, favoring eQTNs. In fact, the prior probabilities of the two models are related by:

$$P(\gamma_j = 1 | d_j = 0) \approx \pi_V, \qquad P(\gamma_j = 1 | d_j = 1) = \pi_G. \tag{39}$$

To see this, we note that under cTWAS, the prior probability of a non-eQTN variant being causal variant to the GWAS trait is π_V. The prior probability of an eQTN being causal variant is just the prior probability that its target gene is causal, which is π_G. We can now express α_1, the key Enloc parameter, in terms of π_G and π_V:

$$\alpha_1 = \log \frac{\pi_G/(1 - \pi_G)}{\pi_V/(1 - \pi_V)} \approx \log \frac{\pi_G}{\pi_V}, \tag{40}$$

where we use the approximation that π_G, π_V are usually small, so $1 - \pi_G \approx 1$ and $1 - \pi_V \approx 1$. There is one subtle difference between this reduced cTWAS model and Enloc. Enloc uses a common parameter for the prior variance of effect sizes for all variants, including eQTNs. cTWAS in contrast, allows the prior variance to differ between gene effects (hence eQTL effects) and variant effects. In our simulations, we have found that using a common prior variance for gene and variant effects leads to inflated PIPs (data not shown).

Having shown the near equivalence of Enloc and the reduced cTWAS model, we can see that cTWAS is also related to coloc [5]. coloc performs model selection, comparing several models, H_1: there is an eQTL in the region being analyzed but no causal variant for GWAS, H_2: there is a causal variant for GWAS trait but no eQTL, H_3: there are distinct eQTL and GWAS causal variants, and H_4: the same causal variant affects both expression and phenotype. The goal of coloc is largely about estimating the posterior probability of H_4, i.e. $P(H_4 | D)$, where D denotes the eQTL and GWAS data we have. This probability is related to the

Enloc model by:

$$P(H_4|D) = \sum_j P(d_j = 1, \gamma_j = 1|D). \tag{41}$$

Having shown the connection between coloc and Enloc, we can also relate the key parameters under the two models. In coloc, the prior probabilities of the four models are related to the prior inclusion probabilities of variants: p_1, the prior of a variant being eQTN; p_2, the prior of causal variant of GWAS trait; and p_{12}, the prior of a variant being causal to both expression and GWAS trait. Whether coloc detects a colocalization, i.e. H_4 is chosen, is controlled largely by these prior parameters, p_1, p_2, p_{12}. In the Enloc paper, the authors show that Enloc is equivalent to coloc with the parameters under the two methods related by (see Equation 10 of Enloc):

$$\alpha_1 = \log \frac{p_{12}(1 - p_1 - p_2 - p_{12})}{p_1 p_2} \approx \log \frac{p_{12}}{p_1 p_2}, \tag{42}$$

where we again assume small prior probabilities. We can also see that the coloc parameteres are related to the cTWAS parameters. In fact, p_1 is the prior of causal variant, so $p_1 \approx \pi_V$; and p_{12}/p_2 is the conditional probability that a variant is causal to the phenotype given that it is an eQTN. As we have explained above, this is $P(\gamma_j = 1|d_j = 1)$, which is π_G under the reduced cTWAS model. So the enrichment parameter under coloc becomes:

$$\log \frac{p_{12}}{p_1 p_2} = \log \frac{p_{12}/p_2}{p_1} \approx \log \frac{\pi_G}{\pi_V}. \tag{43}$$

This is the same Equation 40 we have derived linking α_1 in Enloc and prior parameters of cTWAS.

From these derivations, we see that under the simple scenario of single causal gene, and single eQTN, cTWAS is equivalent to Enloc and coloc. Compared to coloc, both Enloc and the reduced cTWAS have the advantage that all the key prior parameters are estimated from the genome-wide data analysis. In contrast, coloc sets the parameters, p_1, p_2, p_{12}, somewhat arbitrarily. Many have reported that coloc results are sensitive to these parameters. Of course, the equivalence of cTWAS with colocalization methods only applies in the simple scenario. In general, cTWAS has the advantage that it allows multiple causal genes, and multiple eQTNs.

Running other methods on simulation data

We ran FUSION (`https://github.com/gusevlab/fusion_twas`) on the same simulated GWAS summary statistics data and the same gene prediction models. We ran coloc (`https://cran.r-project.org/web/packages/coloc/index.html`) which is provided as part of the FUSION package and used PIP4 > 0.8 as cutoff. We ran FOCUS (`https://github.com/bogdanlab/focus`, v0.6) under the default setting and use PIP > 0.8 as the cut off. For SMR with HEIDI filter (`https://yanglab.westlake.edu.cn/software/smr/`), we used the significant ceQTL marginal association statistics provided by GTEx v7 as input, for genes with FUSION prediction models. These include significant variants $\pm$1mb of the transcription start site (TSS) of each gene. For SMR (v1.0.3), we used B-H adjusted p values < 0.05 to select significant genes, and we also required p_HEIDI > 0.05 for the HEIDI filter. For MR-JTI (`https://github.com/gamazonlab/MR-JTI`), we used the full eQTL marginal association statistics provided by GTEx v7 after LD-pruning ($r^2 = 0.2$) as input. These include all variants $\pm$1mb of the TSS of each gene. We analyzed genes with Fusion B-H adjusted p values < 0.05 using MR-JTI and required a Bonferroni-corrected $p < 0.05$ to determine significance. For PMR-Egger (`https://github.com/yuanzhongshang/PMR`, v1.0), we used the full eQTL marginal association statistics provided by GTEx v7 as input, for genes with FUSION prediction models. We restricted the data to variants $\pm$100kb of the body of each gene. Based on correspondence with the author of PMR-Egger, we regularized the LD matrices for these variants as $0.8\boldsymbol{R} + 0.2\boldsymbol{I}$ to avoid matrix decomposition errors. We used B-H adjusted $p < 0.05$ to select significant genes for PMR-Egger. For MRLocus (v0.0.25), we used the full eQTL marginal association statistics provided by GTEx v7 as input. We restricted our analysis to genes with FUSION B-H adjusted p values < 0.05. For these genes, we used the recommended settings from the MRLocus paper. These include clumping variants with LD $r^2 > 0.1$ and retaining only those with eQTL

$p < 0.001$. After clumping and running the colocalization model, we trimmed eSNPs with LD $r^2 > 0.1$, prioritizing those with the highest eQTL significance. To control for multiple testing, we used a local false sign rate (LFSR) ¡ 0.1s. LFSR is analogous to local false discovery rate (LFDR), but reflects confidence in the sign of effect rather than in the effect being non-zero[6].

Detailed simulation results for PMR-Egger

While all competing methods suffered from high false positive rates in our simulations, PMR-Egger performed particularly poorly. Its false rate rate was even higher than standard TWAS (FUSION). This is quite unexpected, given that the objective of PMR-Egger is to avoid false positives under TWAS. We thus investigated the simulation results for PMR-Egger in more detail to understand the reasons for its poor performance.

We begin with a short review of PMR-Egger. PMR-Egger analyzes one gene a time. Let X be the expression of a gene of interest, and $\tilde{X}$ be its *cis*-genetic component. PMR-Egger assumes that the trait y, depends on $\tilde{X}$, as well as the pleiotropic effect of nearby variants, whose genotypes are denoted as Z_y (p-dimension). We have:

$$y = \mu_y + \tilde{X}\alpha + Z_y\gamma + \epsilon_y, \tag{44}$$

where μ_y is the mean trait value, α is the gene effect, and γ the p-dimensional effect sizes of nearby variants. PMR-Egger also accounts for the uncertainty of $\tilde{X}$ through another model linking observed expression X with genotypes of all nearby variants under a polygenic assumption. The model as shown in Equation 44, by itself, is not identifiable, because of colinearity of $\tilde{X}$ and Z_y: $\tilde{X}$ is effectively a linear combination of variant genotypes. Thus PMR-Egger made an extra assumption, known as Egger regression in Mendelian Randomization, that all variants have identical pleiotropic effects: $\gamma_1 = \gamma_2 = \cdots = \gamma_p = \gamma$. The goal of PMR-Egger is to test if $\alpha = 0$, while allowing $\gamma \neq 0$.

When we first ran PMR-Egger with default settings, we noted that it did not return results for a large percentage of genes (83.4% on average for the high PVE simulations) due to errors decomposing LD matrices. Tuning the shrinkage parameter (λ), as suggested by the software, did not substantially reduce the number of decomposition errors. Upon discussion with the author, we performed an alternative regularization of the LD matrices, regularizing them as $0.8\boldsymbol{R} + 0.2\boldsymbol{I}$, where $\boldsymbol{R}$ is the original LD matrix, and $\boldsymbol{I}$ the identity matrix. The number of decomposition errors was substantially reduced, although a substantial fraction of genes still have errors (18.6% on average for the high PVE simulations).

Among the genes that were significant by PMR-Egger, the proportion of false positives was very high (85.9% on average for the high PVE simulations), higher than FUSION. To understand this discrepancy, we focused on a single simulation from the high PVE scenario and the 6,454 genes which had both FUSION and PMR-Egger results. We observed that $-\log_{10} p$-values for FUSION and PMR-Egger are generally correlated ($r = 0.567$), but there are outlier genes which are insignificant by FUSION but highly significant by PMR-Egger (Fig. S19A). Many of these outlier genes are confounded by nearby causal variants with relatively large effect sizes.

As an example, we visualized the locus containing the gene *ICA1*, which was insignificant by FUSION, but had very significant p-values ($\sim 10^{-29}$) by PMR-Egger (Fig. S19B). At this locus, there is a single causal variant with a large effect size, and no causal gene. As expected, the causal variant shows the strongest association in the locus (Fig. S19B, top, the red vertical bar). The FUSION expression prediction model of *ICA1* has two variants, both of which are nearby (within 72kb) the causal variant (Fig. S19B, middle), but are not in LD with the causal variant ($R^2 = -0.022$ for strongest correlation). In the eQTL analysis of *ICA1*, the two variants in the prediction model show strong associations, as expected, but the causal variant is also nominally significant (Fig. S19B, bottom). Because PMR-Egger uses all *cis*-variants of a gene to predict expression under a polygenic assumption, it is likely that the prediction model of PMR-Egger includes some contribution of the causal variant. This induces a correlation of predicted expression of *ICA1* with the simulated trait. On the other hand, the null model of PMR-Egger, where $\alpha = 0$, attempts to explain the data using an identical effect for all *cis*-variants of *ICA1*. However, this is a mismatch of the data, as the association is simply due to a single causal variant with large effect. Taken together, we believe

these two facts (the correlation of $\tilde{x}$ and y due to PMR-Egger's expression prediction model, and the poor fit of the data by the null model) explain the false positive finding by PMR-Egger in this case. The FUSION method, on the other hand, avoided this mistake. The two variants of *ICA1* are not in high LD with the causal variant, and have low associations with the trait in GWAS (Fig. S19B, top). As a result, the imputed expression using FUSION is not highly correlated with the trait.

Many other genes that are discordant between FUSION and PMR-Egger are qualitatively similar. These results suggest that PMR-Egger is susceptible to false positives, when (1) the underlying expression prediction model is sparse with only a few causal variants, and (2) when there is a nearby large effect causal variant acting on the phenotype directly.

Prediction model of gene expression.

We used expression prediction models for 49 GTEx [7] tissues from PredictDB [8] [3]. We used the mashr-based prediction models, which borrowed information across tissues to improve prediction accuracy in any specific tissue [9]. These prediction models were built using fine-mapped variants and are sparse, with a maximum of 5 eQTL per gene. The models were constructed using in-sample LD from GTEx, and the covariance between pairs of variants within each gene is reported alongside the models. We included only protein-coding genes for our analyses. Variants included in the models but missing in the GWAS summary statistics or LD reference were given zero weight.

Total heritability and percentage of heritability mediated by genes using MESC.

To assess parameter estimates from cTWAS, we computed total heritability and the percentage of heritability mediated by genes using MESC [10] for comparison. We used the same summary statistics as in the cTWAS analysis, and we used GTEx v8 expression scores for individual tissues available via the MESC repository. These expression scores are derived from the same GTEx dataset as the PredictDB models. Note that expression scores for "Kidney Cortex" tissue were not available from MESC, although this tissue is included in PredictDB and our other analyses.

Silver standard and previously reported candidate genes of complex traits.

To evaluate our findings, we compared the genes detected using cTWAS to curated lists of known or candidate genes for each trait. For LDL, we combined curated gene lists from two previous studies [11] [12], for a total of 69 LDL-related genes. These are genes that cause Mendelian disease or are drug targets, are in the KEGG "cholesterol metabolism" pathway, or are otherwise documented in the literature. For IBD, we used a list of 26 genes curated from literature, reported in the paper describing the activity-by-contact (ABC) model [13]. We refer to the gene lists for LDL and IBD as "silver standard" gene lists throughout the text based on the strength of their evidence. In supplemental materials, we also refer to "previously reported candidate genes" for SCZ and SBP. These are gene lists that are primarily supported by GWAS data rather than literature or experimental evidence. For SCZ, we used a list of 120 genes prioritized as SCZ-related based on proximity to GWAS, fine-mapping results and eQTL evidence [14]. For SBP, we used a list of 53 genes, supported by TWAS, gene expression, drug targets, pathway analysis and rare variant studies, reported in a large trans-ethnic GWAS (Table 3 of the paper) [15].

Assessing the novelty of cTWAS detected genes.

If a gene was not included in the list of silver standard or previously reported genes of a trait, and was not the nearest gene of the lead variant in a genome-wide significant locus, we considered the gene a "novel" finding. We defined "nearest" genes as those with start or end positions that were the nearest out of all protein-coding genes to the lead variant of a genome-wide significant locus. To define independent lead variants for the GWAS, we performed the following procedure. First, we selected the most significant

variant as a lead variant, and we removed all other variants within 500kb. Then, we iterated these selection and removal steps until there were no genome-wide significant variants remaining. We then identified the protein-coding genes that were nearest to each lead variant in the resulting list.

Gene Ontology (GO) analysis of candidate genes.

We performed enrichment analysis to characterize the genes detected by cTWAS for LDL and IBD. We used Enrichr [16] to identify Gene Ontology terms in the Biological Process, Molecular Function, or Cellular Component domains that are enriched for genes detected by cTWAS. Enrichr uses the Benjamini-Hochberg procedure to control FDR in each domain. We used a threshold of FDR ¡ 0.05 for significant enrichment. We report the full enrichment analysis results using Enrichr across all domains for LDL and IBD (Supplementary Table 3; Supplementary Table 10). We compared the results from cTWAS genes with similar enrichment analysis results that used silver standard genes for LDL and IBD (Supplementary Table 4; Supplementary Table 11). We also compared these results with enrichment results from MAGMA [17] [18], a method that performs enrichment analysis based on GWAS data, using default settings (Supplementary Table 5; Supplementary Table 12). When reporting the IBD results (Supplementary Table 8), we annotated detected genes with the enriched GO terms (using cTWAS genes, silver standard genes, or MAGMA) that each gene is a member of. For all three GO annotations, a maximum of 5 significant terms per gene were shown, ordered by odds ratios (cTWAS, silver standard) or p-values (MAGMA).

To visualize the GO biological process terms enriched for LDL cTWAS genes (Fig. 5b), we removed redundant terms to improve clarity. To do this, we first ranked all GO terms by their significance (p-values). A term was considered redundant if all the cTWAS genes in that term had already been included in a more significant GO term.

To visualize the GO biological process terms enriched for IBD cTWAS genes (Fig. 6e), we used the ”Weighted Set Cover” tool in WebGestalt [19] to remove redundant GO terms. To further simplify visualization, we omitted GO terms whose detected genes were all included in other terms identified by Weighted Set Cover. We also report the full enrichment analysis results using WebGestalt for IBD (Supplementary Table 9).

Analyzing the HPR and POLK / HMGCR loci using other methods.

We analyzed the genes at the HPR and POLK loci using coloc, SMR, and FOCUS. We applied these methods as described in the ”Running other methods on simulation data” subsection of the methods, with the following exception. For SMR, rather than use a FDR-based significance threshold, which requires p-values genome-wide, we used a local Bonferroni significance threshold to account for the number of genes tested at each locus.

Fine-mapping analysis of POLK / HMGCR locus.

To examine the POLK / HMGCR locus, we used fine-mapping results of LDL from UK Biobank from PolyFun-SuSiE [20] (downloaded from `http://data.broadinstitute.org/alkesgroup/polyfun_results/biochemistry_LDLdirect.txt.gz`). Specifically, PolyFun specifies prior probabilities for fine-mapping by estimating functional enrichments for a broad set of coding, conserved, regulatory and LD-related annotations. Then SuSiE was applied using the estimated priors with L=10 effects per locus.

To annotate the functions of variants their putative target genes, we used additional datasets. H3K4me1 ChIP-seq data of from human liver samples were downloaded from the ENCODE portal (ENCODE ID: ”ENCFF323TFF”). Promoter Capture Hi-C (PC-HiC) data from human liver were obtained from a previous study [21]. Liver activity-by-contract (ABC) data were also obtained from a previous study [22].

Novel candidate genes from application of cTWAS on several complex traits.

The application of cTWAS to several complex traits suggested interesting and novel candidate genes. In the case of LDL, we identified two genes, ACVR1C and INHBB, in the signaling pathway of activin, a signaling molecule in the TGF-beta superfamily. ACVR1C is a receptor of activin A and INHBB is a subunit of activin B. Both activin A and B regulate lipolysis in hepatocytes and adipocytes and alter lipid compositions [23] [24]. In an exome-wide association study, a loss-of-function variant of ACVR1C was associated with metabolic phenotypes such as triglycerides and high-density lipoprotein level [25]. We identified another signal transduction gene, a protein kinase, PRKD2, as a candidate gene affecting LDL. PRKD2 deficiency in mice triggers hyperinsulinemia, metabolic disorders and dysregulation of LDL [26].

Our study of IBD revealed a number of interesting candidate IBD risk genes (Table 1). IFNGR2 is a receptor of interferon-gamma, a proinflammatory cytokine, whose activation may result in intestinal lesions [27]. IRF8 is an important regulator of multiple immune processes ranging from antigen presentation to response to cytokines [28]. IRF8 deficiency in an experimental model of colitis resulted in more severe inflammation [29]. CCR5 is a chemokine receptor, best known for its role in HIV infection. CCR5 blockade in mice inhibited leukocyte trafficking and reduced mucosal inflammation in murine colitis [30]. TYMP encodes thymidine phosphorylase, and TYMP mutations cause Mitochondrial Neurogastrointestinal Encephalomyopathy (MNGIE), a rare recessive disease [31]. The disease causes gastrointestinal symptoms and is often misdiagnosed as celiac or Crohn's disease [31]. LSP1 is a regulator of T-cell migration, and deletion of LSP1 gene has been implicated in rheumatoid arthritis, an autoimmune disease [32].

References

[1] Jon Wakefield. "Bayes factors for genome-wide association studies: comparison with P-values". In: *Genetic Epidemiology: The Official Publication of the International Genetic Epidemiology Society* 33.1 (2009), pp. 79–86.

[2] Xiaoquan Wen et al. "Efficient integrative multi-SNP association analysis via deterministic approximation of posteriors". In: *The American Journal of Human Genetics* 98.6 (2016), pp. 1114–1129.

[3] Barbeira A. "Exploring the phenotypic consequences of tissue specific gene expression variation inferred from GWAS summary statistics". In: *Nat. Commun* 9 (2018), p. 1825.

[4] Wen X, Pique-Regi R, and Luca F. "Integrating molecular QTL data into genome-wide genetic association analysis: Probabilistic assessment of enrichment and colocalization". In: *PLoS Genet* 13 (2017), e1006646.

[5] Hormozdiari F. "Colocalization of GWAS and eQTL Signals Detects Target Genes". In: *Am. J. Hum. Genet* 99 (2016), pp. 1245–1260.

[6] Matthew Stephens. "False discovery rates: a new deal". In: *Biostatistics* 18.2 (Oct. 2016), pp. 275–294.

[7] "The Genotype-Tissue Expression (GTEx) project". In: *Nat. Genet* 45 (2013), pp. 580–585.

[8] Gamazon E. "A gene-based association method for mapping traits using reference transcriptome data". In: *Nat. Genet* 47 (2015), pp. 1091–1098.

[9] Barbeira A. "Integrating predicted transcriptome from multiple tissues improves association detection". In: *PLoS Genet* 15 (2019), e1007889.

[10] Yao D et al. "Quantifying genetic effects on disease mediated by assayed gene expression levels". In: *Nat. Genet* 52 (2020), pp. 626–633.

[11] Zhou D. "A unified framework for joint-tissue transcriptome-wide association and Mendelian randomization analysis". In: *Nat. Genet* 52 (2020), pp. 1239–1246.

[12] Forgetta V. "An effector index to predict target genes at GWAS loci". In: *Hum. Genet* 141 (2022), pp. 1431–1447.

[13] Lee A and Jung I. "Functional annotation of lung cancer-associated genetic variants by cell typespecific epigenome and long-range chromatin interactome". In: *Genomics Inform* 19 (2021), e3.

[14] Trubetskoy V. "Mapping genomic loci implicates genes and synaptic biology in schizophrenia". In: *Nature* 604 (2022), pp. 502–508.

[15] Giri A. "Trans-ethnic association study of blood pressure determinants in over 750,000 individuals". In: *Nat. Genet* 51 (2019), pp. 51–62.

[16] Chen E. "Enrichr: interactive and collaborative HTML5 gene list enrichment analysis tool". In: *BMC Bioinformatics* 14 (2013), p. 128.

[17] De Leeuw C et al. "MAGMA: generalized gene-set analysis of GWAS data". In: *PLoS Comput. Biol* 11 (2015), e1004219.

[18] Watanabe K et al. "Functional mapping and annotation of genetic associations with FUMA". In: *Nat. Commun* 8 (2017), p. 1826.

[19] Liao Y et al. "WebGestalt 2019: gene set analysis toolkit with revamped UIs and APIs". In: *Nucleic Acids Res* 47 (2019), W199–W205.

[20] Weissbrod O. "Functionally informed fine-mapping and polygenic localization of complex trait heritability". In: *Nat. Genet* 52 (2020), pp. 1355–1363.

[21] Jung I. "A compendium of promoter-centered long-range chromatin interactions in the human genome". In: *Nat. Genet* 51 (2019), pp. 1442–1449.

[22] Nasser J. "Genome-wide enhancer maps link risk variants to disease genes". In: *Nature* 593 (2021), pp. 238–243.

[23] Yndestad A. "A complex role of activin A in non-alcoholic fatty liver disease". In: *Am. J. Gastroenterol* 104 (2009), pp. 2196–2205.

[24] Yogosawa S et al. "Activin receptor-like kinase 7 suppresses lipolysis to accumulate fat in obesity through downregulation of peroxisome proliferator-activated receptor γ and C/EBPα". In: *Diabetes* 62 (2013), pp. 115–123.

[25] Koprulu M. "Identification of Rare Loss-of-Function Genetic Variation Regulating Body Fat Distribution". In: *J. Clin. Endocrinol. Metab* 107 (2022), pp. 1065–1077.

[26] Xiao Y. "Deficiency of PRKD2 triggers hyperinsulinemia and metabolic disorders". In: *Nat. Commun* 9 (2018), p. 2015.

[27] Langer V. "IFN-γ drives inflammatory bowel disease pathogenesis through VE-cadherindirected vascular barrier disruption". In: *J. Clin. Invest* 129 (2019), pp. 4691–4707.

[28] Sandra Salem, David Salem, and Philippe Gros. "Role of IRF8 in immune cells functions, protection against infections, and susceptibility to inflammatory diseases". en. In: *Hum. Genet.* 139.6-7 (June 2020), pp. 707–721.

[29] Ouyang X. “Transcription factor IRF8 directs a silencing programme for TH17 cell differentiation”. In: *Nat. Commun* 2 (2011), p. 314.

[30] Mencarelli A. “Highly specific blockade of CCR5 inhibits leukocyte trafficking and reduces mucosal inflammation in murine colitis”. In: *Sci. Rep* 6 (2016), p. 30802.

[31] Kučerová L. “Mitochondrial neurogastrointestinal encephalomyopathy imitating Crohn’s disease: a rare cause of malnutrition”. In: *J. Gastrointestin. Liver Dis* 27 (2018), pp. 321–325.

[32] Hwang S.-H. “Leukocyte-specific protein 1 regulates T-cell migration in rheumatoid arthritis”. In: *Proc. Natl. Acad. Sci. U. S. A* 112 (2015), E6535–6543.

Supplemental Figures

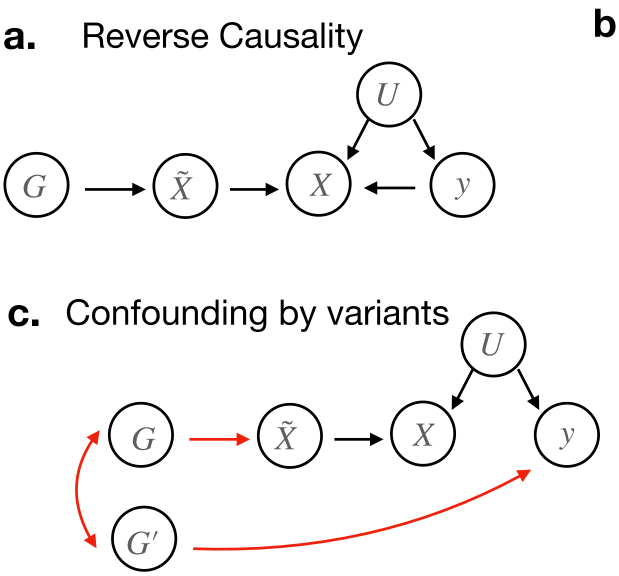

Supplementary Figure 1. Causal diagrams representing several scenarios of how genetic variant, expression and phenotype are related. X and X', gene expression; G and G' are genotypes of variants affecting expression of the two genes; $\tilde{X}$ and $\widetilde{X}'$, *cis*-genetic component of gene expression (*i.e.* imputed expression); y, phenotypic trait; U, unmeasured confounder. Arrows indicate causal effect; double-headed arrows between G and G' indicate LD. Note that it is possible that G and G' are identical variants. This happens when the same variant affects both X and X'. **a.** Reverse causality: the phenotype y has an effect on the expression of gene X. There are two paths from $\tilde{X}$ to y, either through X or not. But in both paths, X is a collider that blocks the association of $\tilde{X}$ and y. **b.** Confounding by a nearby gene X' creates correlation of $\tilde{X}$ and y. The LD between eQTLs of the two genes creates a non-causal, backdoor path (shown in red) between $\tilde{X}$ and y. **c.** Confounding by a nearby variant G' creates correlation of $\tilde{X}$ and y. The backdoor path from $\tilde{X}$ to y through G' is shown in red.

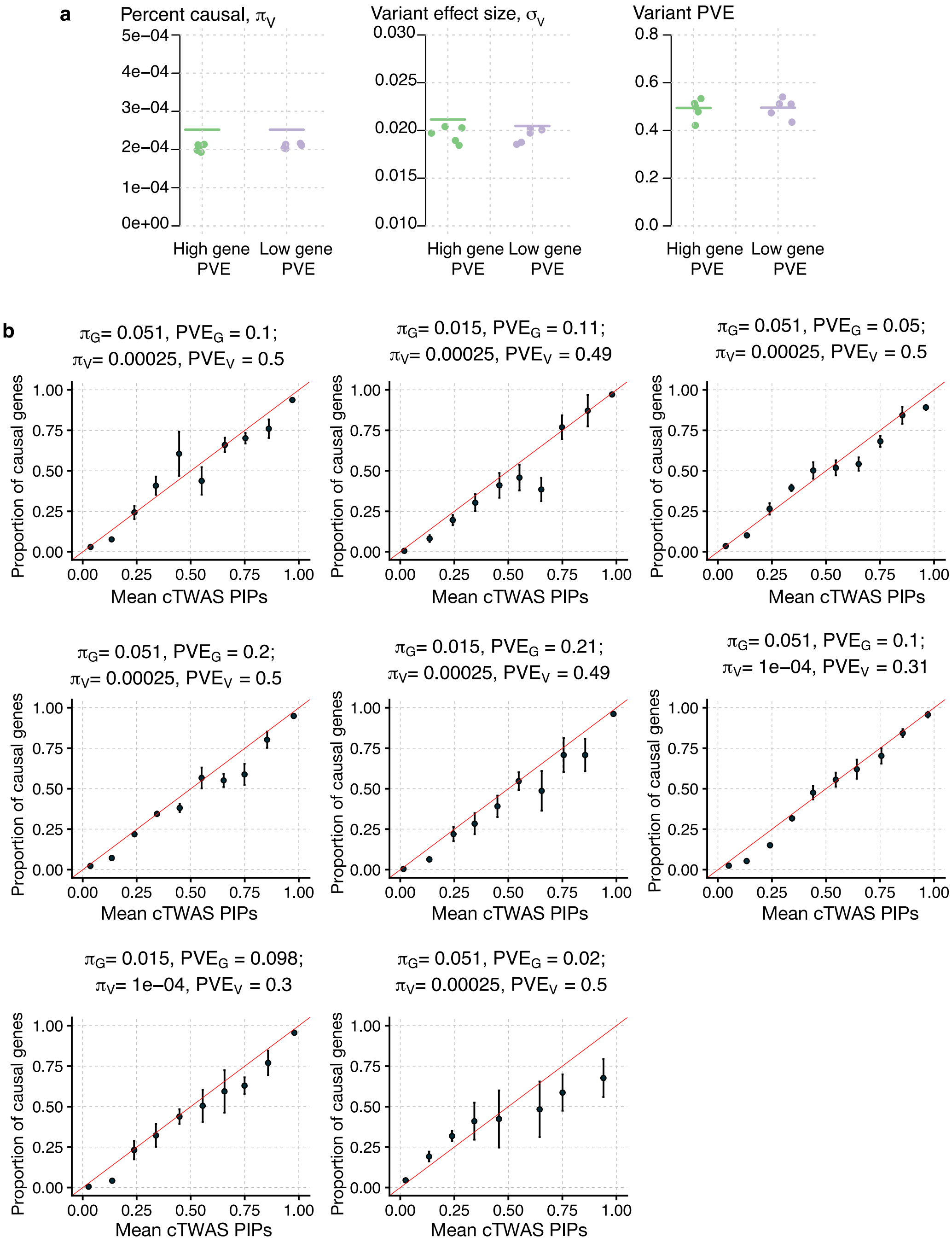

Supplementary Figure 2. Additional simulation results. a. Parameter estimation results for variant effect related parameters. From left to right, percentage of causal variants (π_V), average effect size of causal variants and PVE from variants. The high and low PVE settings are the same as described in Figure 2. **b.** Gene PIP calibration plots under additional settings. For each plot, we listed the parameters used in simulating the trait at the top. π_G is prior probability for a gene being causal, PVE_G is PVE by gene effect; π_V is prior probability for a variant being causal, PVE_V is PVE by variant effect. Under each setting, gene PIPs from 5 simulations are grouped into bins. The plot shows the proportion of true causal genes (Y axis) against the average PIPs (X axis) under each bin. A well calibrated method should produce points along the diagonal lines (red). +/- standard error is shown for each point in the vertical bars calculated over five independent simulations.

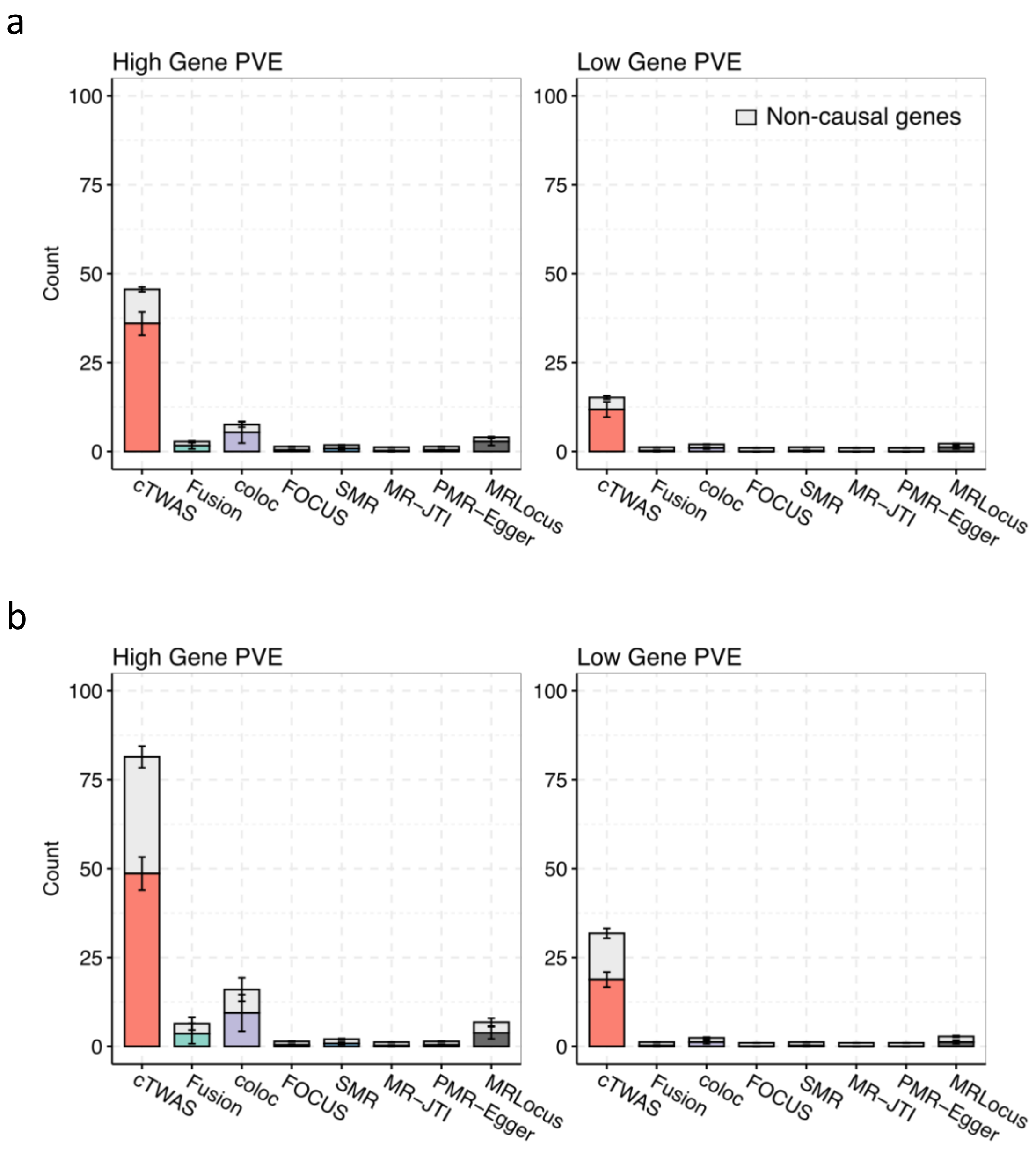

Supplementary Figure 3. Comparison of power at a given false discovery proportion (FDP). Here , FDP is defined as the proportion of false genes among those predicted by a method at a given cutoff. For each method, we select genes by starting from the top of ranked gene lists generated by that method and stop when the given false discovery proportion was reached, then we count the number of causal genes that were found. The height of the bar is the mean over five independent simulations; the standard error is shown for each bar in vertical lines from the same five simulations. **a** and **b**. False discovery proportion 20%. **c** and **d**. False discovery proportion 40%. **a** and **c**, high gene PVE setting as in Figure 2. **b** and **d**, low gene PVE setting as in Figure 2. Given the generally high false positive rates, alternative methods other than cTWAS often cannot reach FDP at 5% or 10%, no matter how stringent the thresholds are, so we compare the methods at FDP cutoff of 20% and 40%.

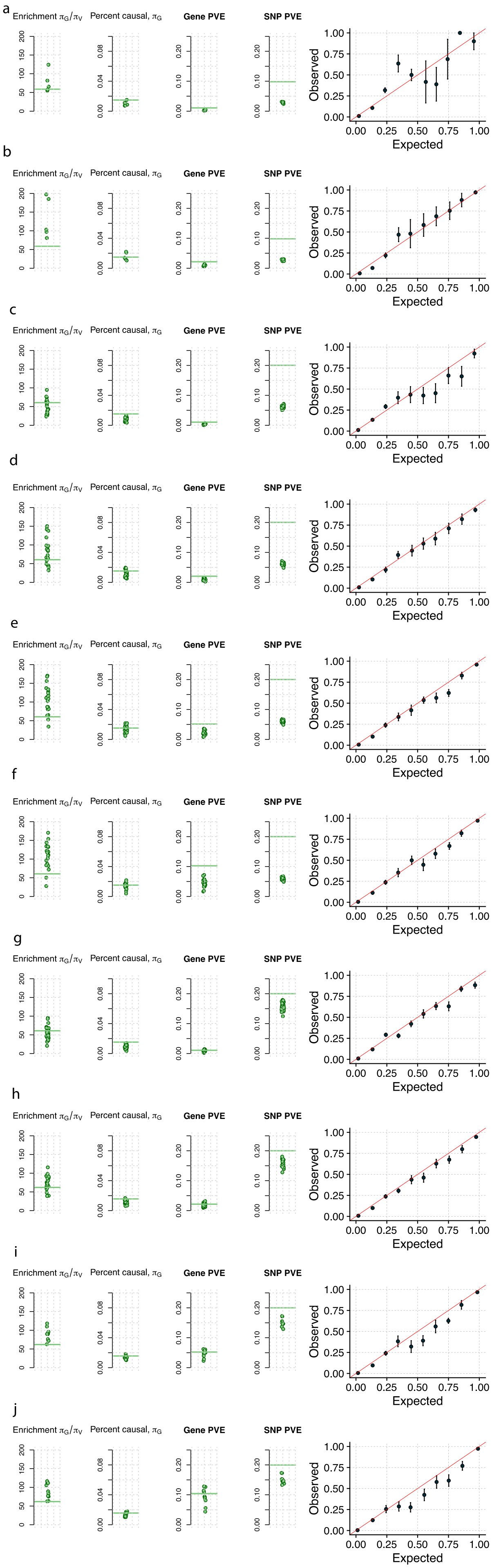

Supplementary Figure 4. Simulations under the low heritability setting. PVE of variants range from 0.1 - 0.2, and PVE of genes from 0.01 to 0.1. From a to j, each panel shows results from one simulation setting. a-f, simulations performed using GWAS with ~45k individuals as described in "Methods". g-j, simulations performed using GWAS with ~113k individuals. We randomly selected 200k individuals from UK Biobank and used the same filtering strategy as described in "Methods" to generate genotype data, which ended up with 112,824 individuals and 6,227,963 variants. The first 4 figures in each panel show parameter estimation results. π_G is the prior probability for a gene being causal; π_V is the prior probability for a SNP being causal; enrichment is defined as π_G/π_V; gene PVE and variant PVE are the percent of phenotypic variance explained by genes and variants, respectively. Each dot represents the result from one out of five simulations. Horizontal bars show the true parameter values. The last figure under each simulation setting is the PIP calibration plot, similarly as described in Figure 2b and Supplementary Figure 2b. Under each setting, gene PIPs from 5 simulations are grouped into bins. The plot shows the proportion of true causal genes (Y axis) against the average PIPs (X axis) under each bin. +/- standard error is shown for each point in the vertical bars calculated over five independent simulations.

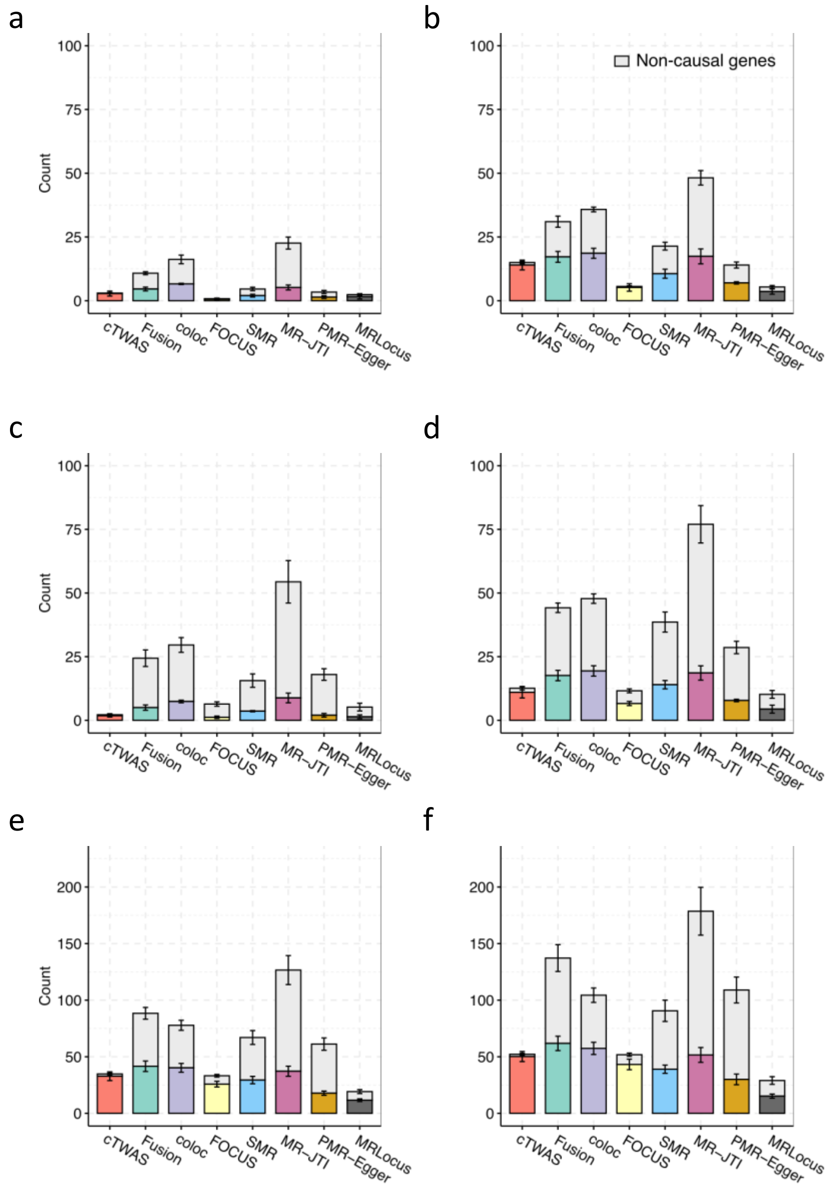

Supplementary Figure 5. Comparison of cTWAS with other methods for low heritability settings. a to f correspond to the simulation settings in supplementary Figure 4a to f. We run comparator methods using their default settings same as described for Figure 3b. We use different colors for causal genes identified by each method, and the top gray bars indicate non-causal genes. The height of the bar is the mean over five independent simulations; the standard error is shown for each bar in vertical lines from the same five simulations.

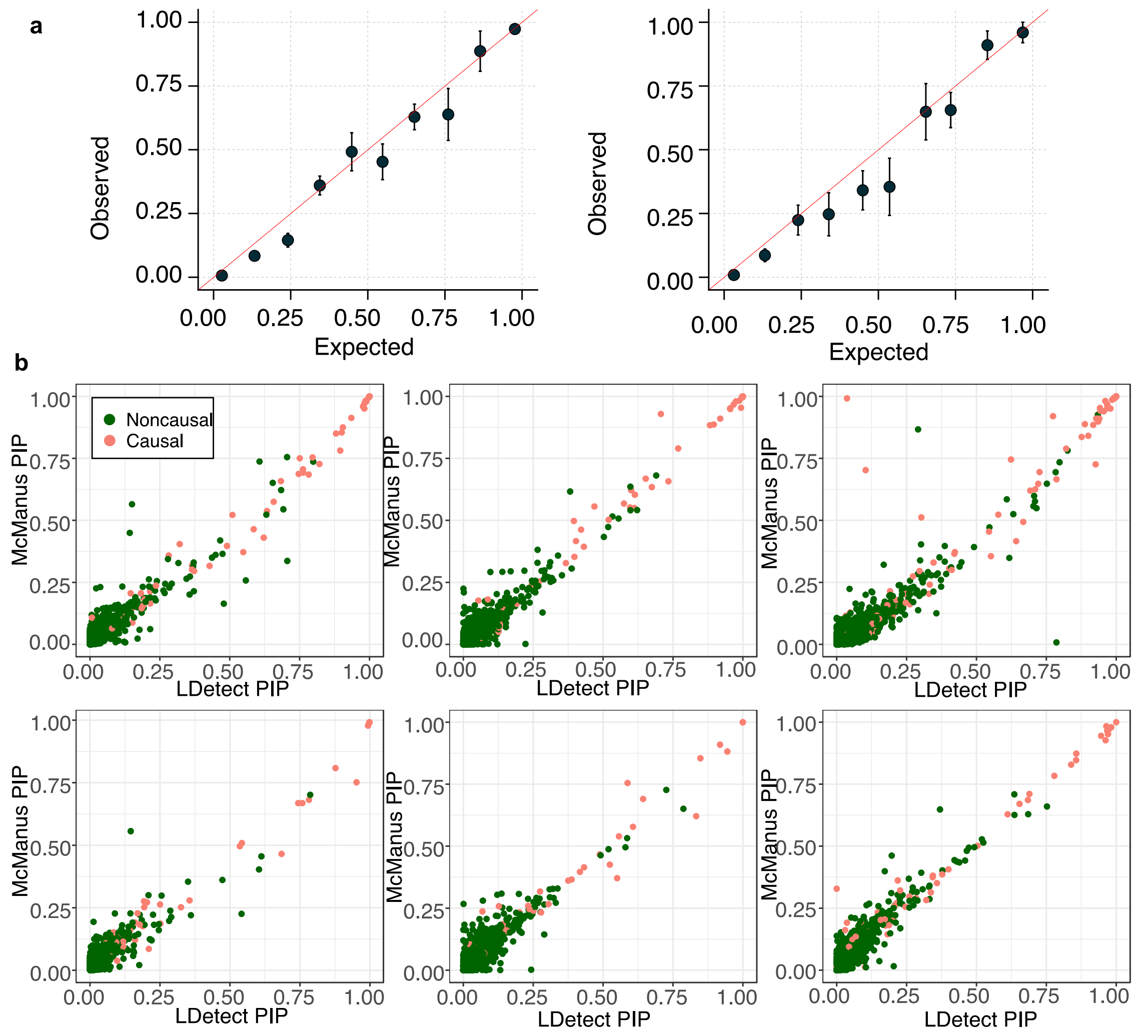

Supplementary Figure 6. PIPs from simulations with the new LD block definition from McManus et al.
a. PIP calibration plots. Left, high gene PVE setting; right, low gene PVE setting, the same settings as in Figure 2. Under each setting, gene PIPs from 5 simulations are grouped into bins. The plot shows the proportion of true causal genes (Y axis) against the average PIPs (X axis) under each bin. +/- standard error is shown for each point in the vertical bars calculated over five independent simulations. b. Scatter plots for PIPs of genes from LDetect blocks (X-axis) and PIPs from McManus blocks (Y-axis). Top row, results from three simulation runs from the high gene PVE setting. Bottom, results from three simulation runs from the low gene PVE setting.

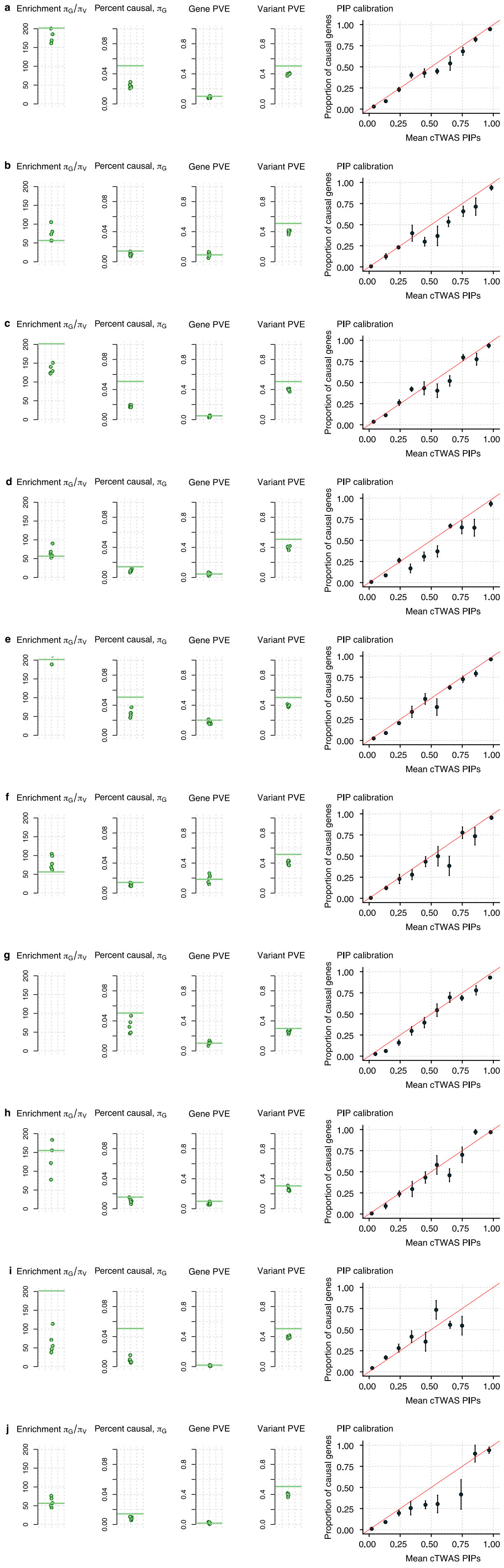

Supplementary Figure 7. Results from simulations using mixtures of normal distribution as the prior distribution of effect sizes. From a to j, each panel shows results from one simulation setting. The first 4 figures in each panel show parameter estimation results. π_G is the prior probability for a gene being causal; π_V is the prior probability for a SNP being causal; enrichment is defined as π_G/π_V; gene PVE and variant PVE are the percent of phenotypic variance explained by genes and variants, respectively. Each dot represents the result from one out of five simulations. Horizontal bars show the true parameter values. The last figure under each simulation setting is the PIP calibration plot, similarly as described in Figure 2b and Supplementary Figure 2b. Under each setting, gene PIPs from 5 simulations are grouped into bins. The plot shows the proportion of true causal genes (Y axis) against the average PIPs (X axis) under each bin. +/- standard error is shown for each point in the vertical bars calculated over five independent simulations.

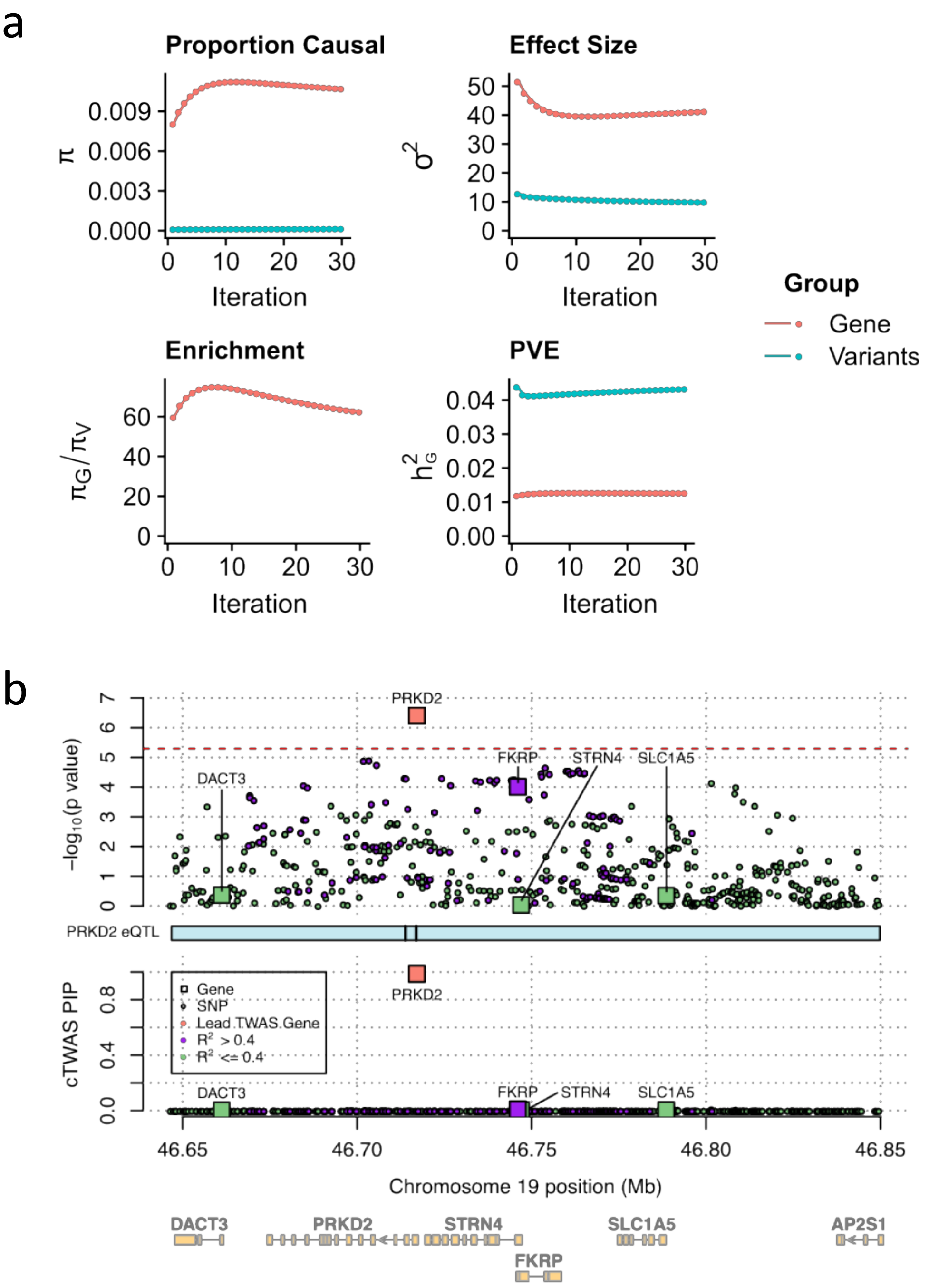

Supplementary Figure 8. Additional results for cTWAS analysis of LDL cholesterol using liver eQTLs. **a.** Convergence of estimated parameters over 30 iterations of the E-M algorithm. “Proportion Causal” is the prior inclusion probability of genes and variants. “Effect Size” is the prior variance of the gene and variant effect sizes. “Enrichment” is the ratio of gene to variant prior inclusion probabilities. “PVE” is the proportion of variance explained by genes and variants. **b.** cTWAS results at the *PRKD2* locus. Figure legend is the same as in the locus plots in the main figures, see Fig. 4b.

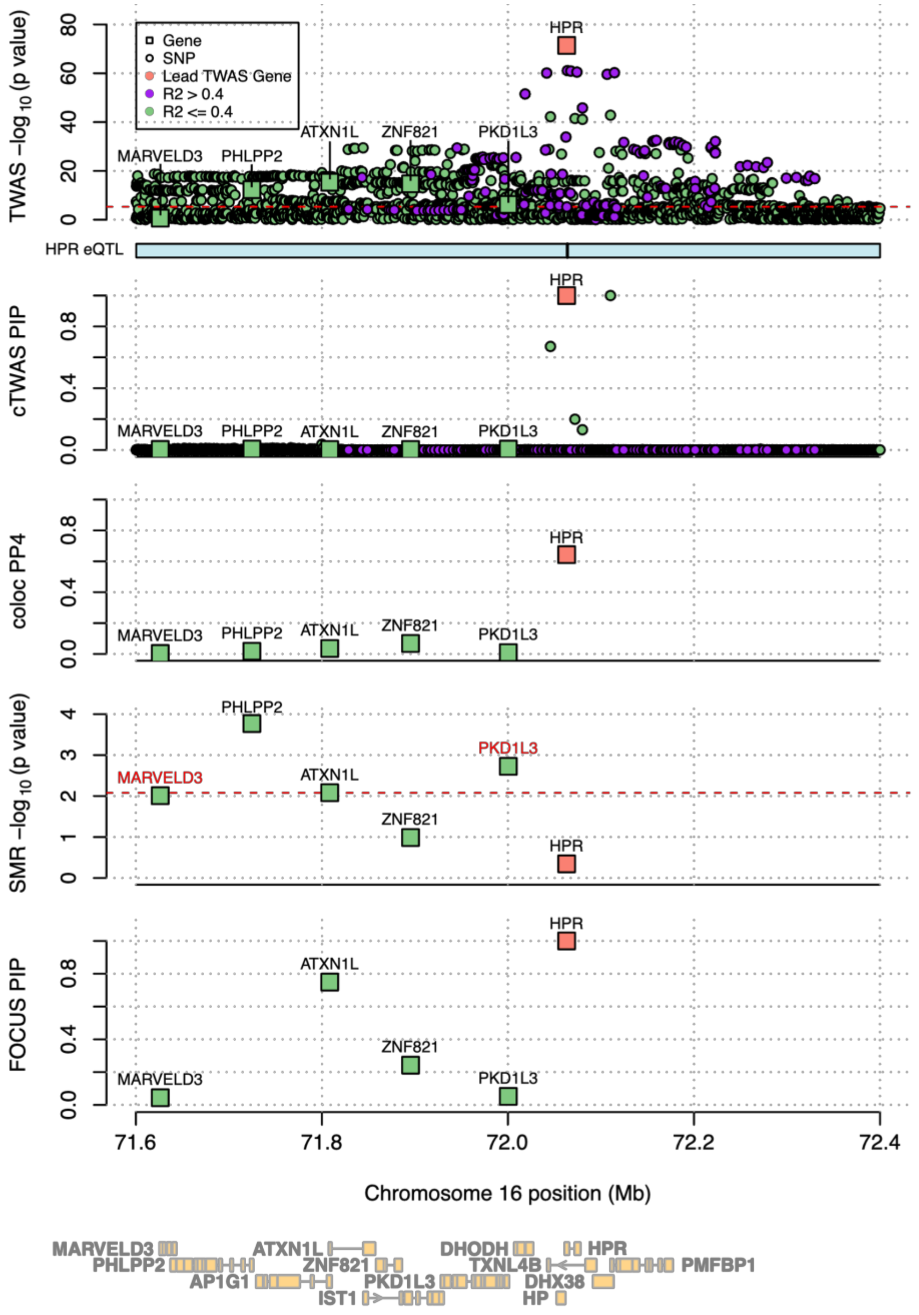

Supplementary Figure 9. Extended results at the *HPR* locus. Figure legend for the top three tracks and the bottom track are the same as in Figure 4b. The fourth track from the top represents the posterior probability of colocalization (PP4) for each gene from coloc. The fifth track depicts the -$\log_{10}$ p-value for each gene using SMR. The red dotted line indicates the local significance threshold for this locus (Bonferroni corrected *p*-value < 0.05 for the genes depicted). Genes labels are colored in red if they do not pass the HEIDI filter. The sixth track shows the PIP for genes at this locus using FOCUS.

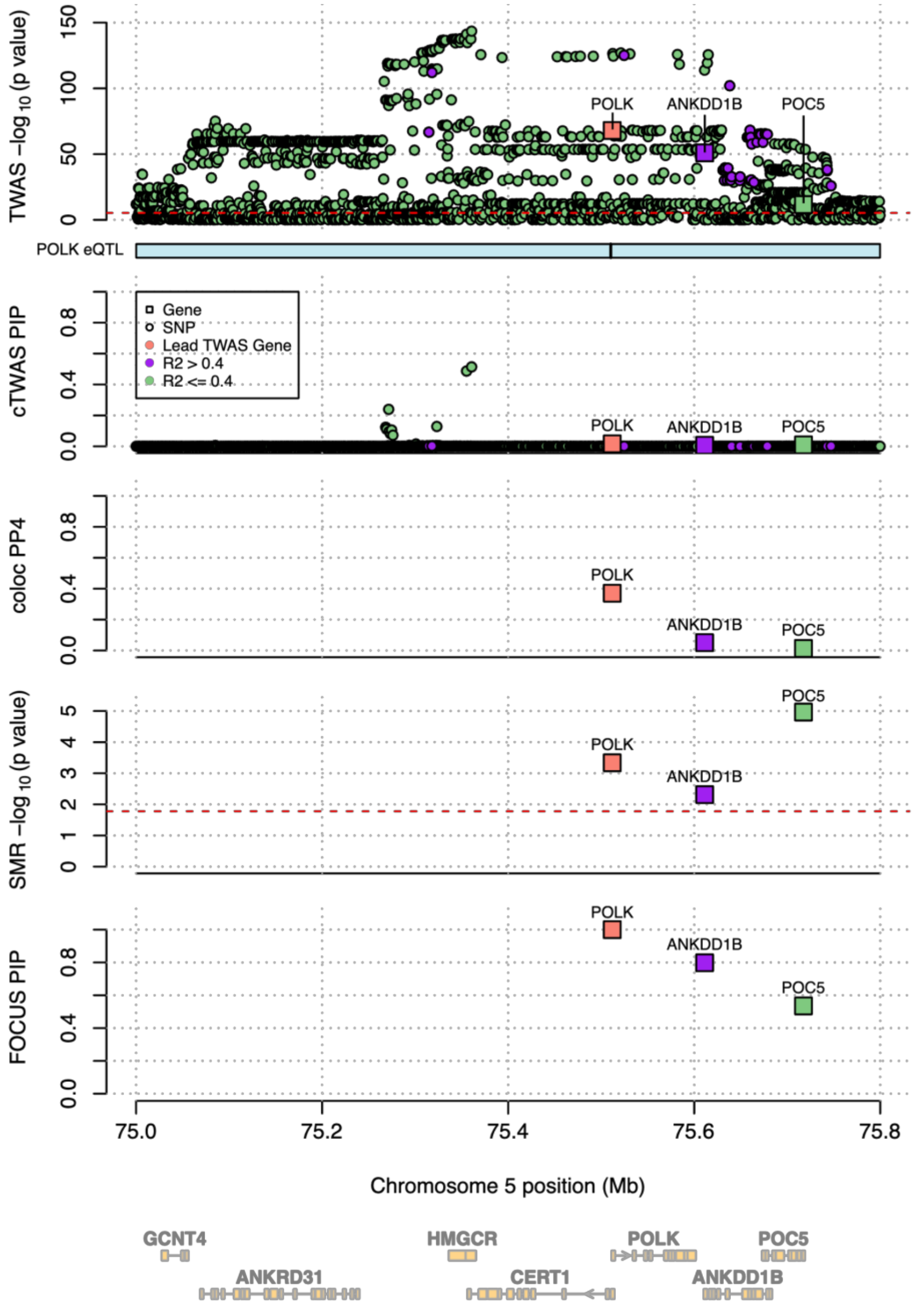

Supplementary Figure 10. Extended results at the *POLK* locus. Same legend as in Supplementary Figure 9.

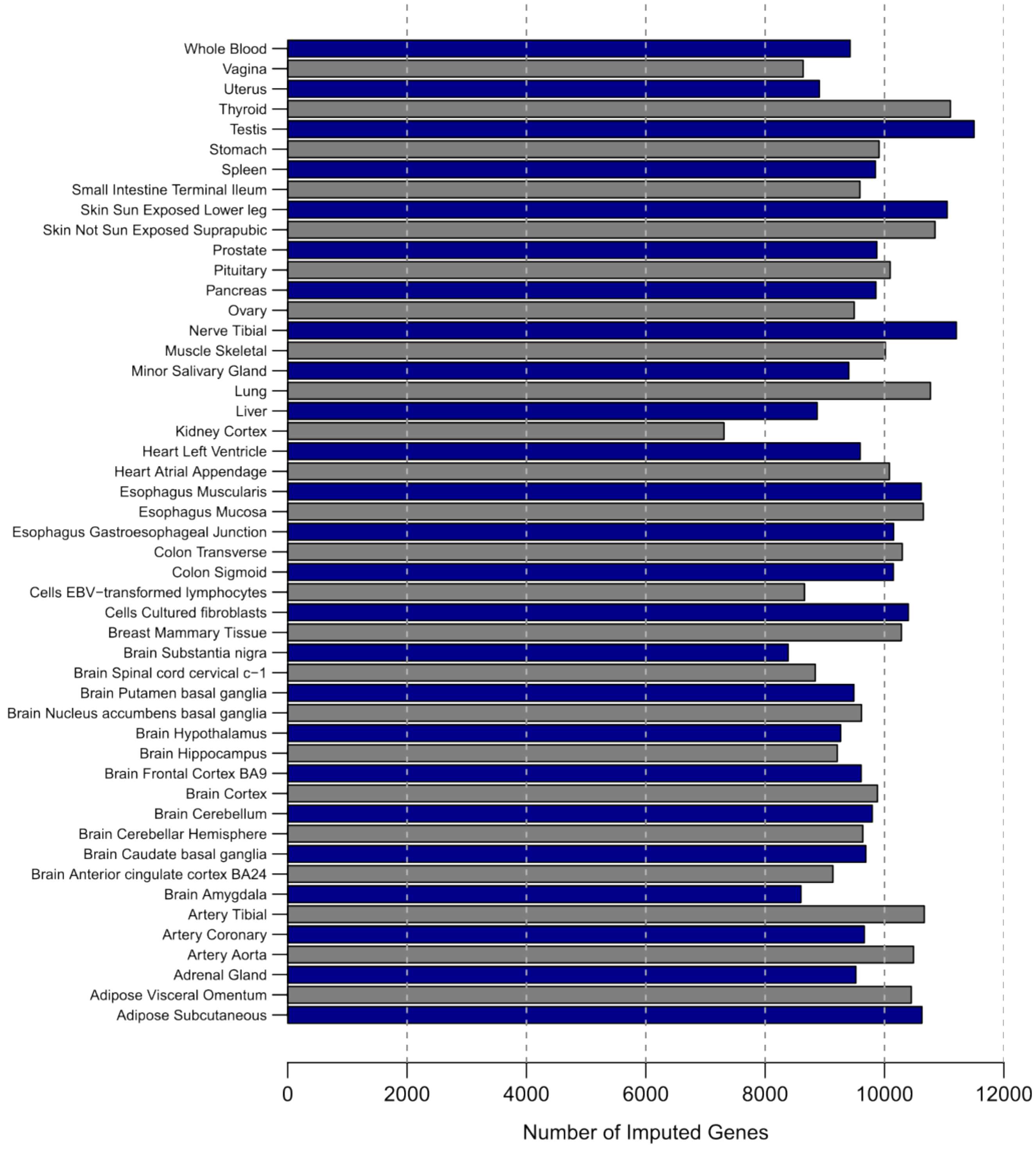

Supplementary Figure 11. Mean number of imputed genes across all tissues for IBD, SCZ and SBP. The numbers of imputed genes analyzed by cTWAS vary slightly among the three traits, because of the differences in the variants included in GWAS summary statistics. For simplicity, we show the mean numbers among three traits.

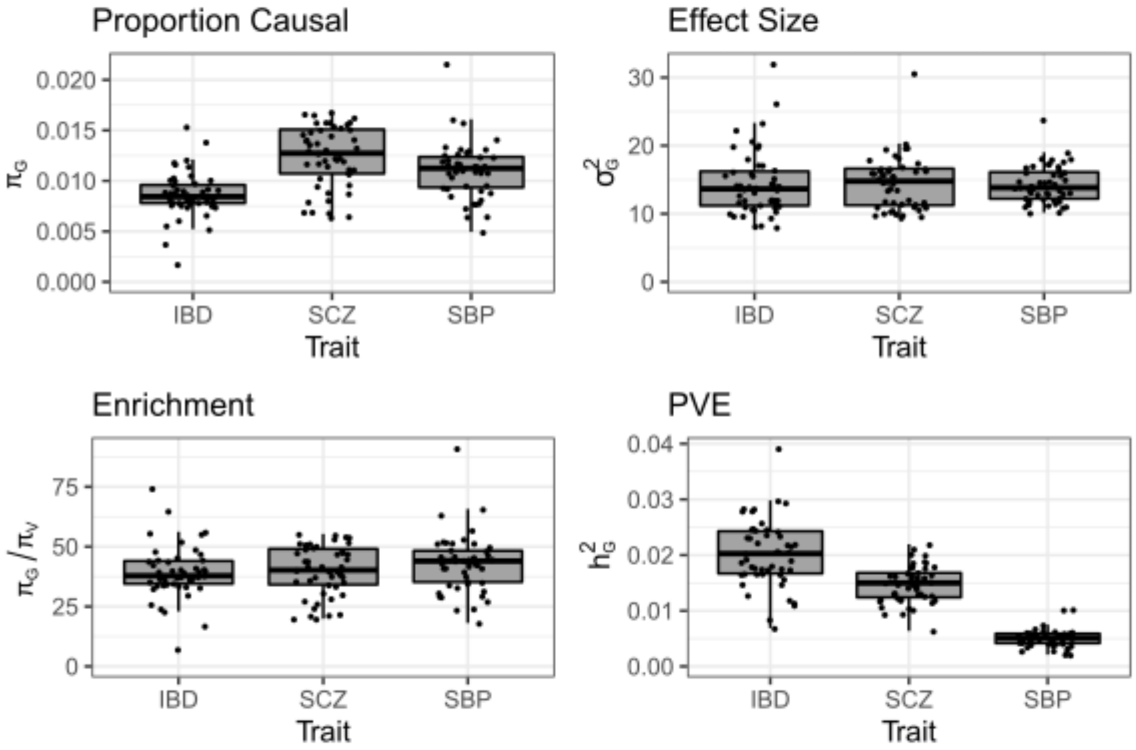

Supplementary Figure 12. Estimated prior parameters for genes across all tissues for IBD, SCZ and SBP. The estimated prior parameters for genes across all n=49 independent GTEx tissues for IBD, SCZ and SBP. Each dot represents the gene parameter for one of the tissues. “Proportion Causal” is the prior inclusion probability of genes. “Effect Size” is the prior variance for gene effects. “Enrichment” is the ratio of gene to variant prior inclusion probabilities. “PVE” is the proportion of variance explained by genes. The centers of the box plots denote medians, the hinges of the boxes denote the 25th and 75th percentiles, and the whiskers extend 1.5 times the inter-quartile range from the hinges, or to the most extreme point, whichever is less.

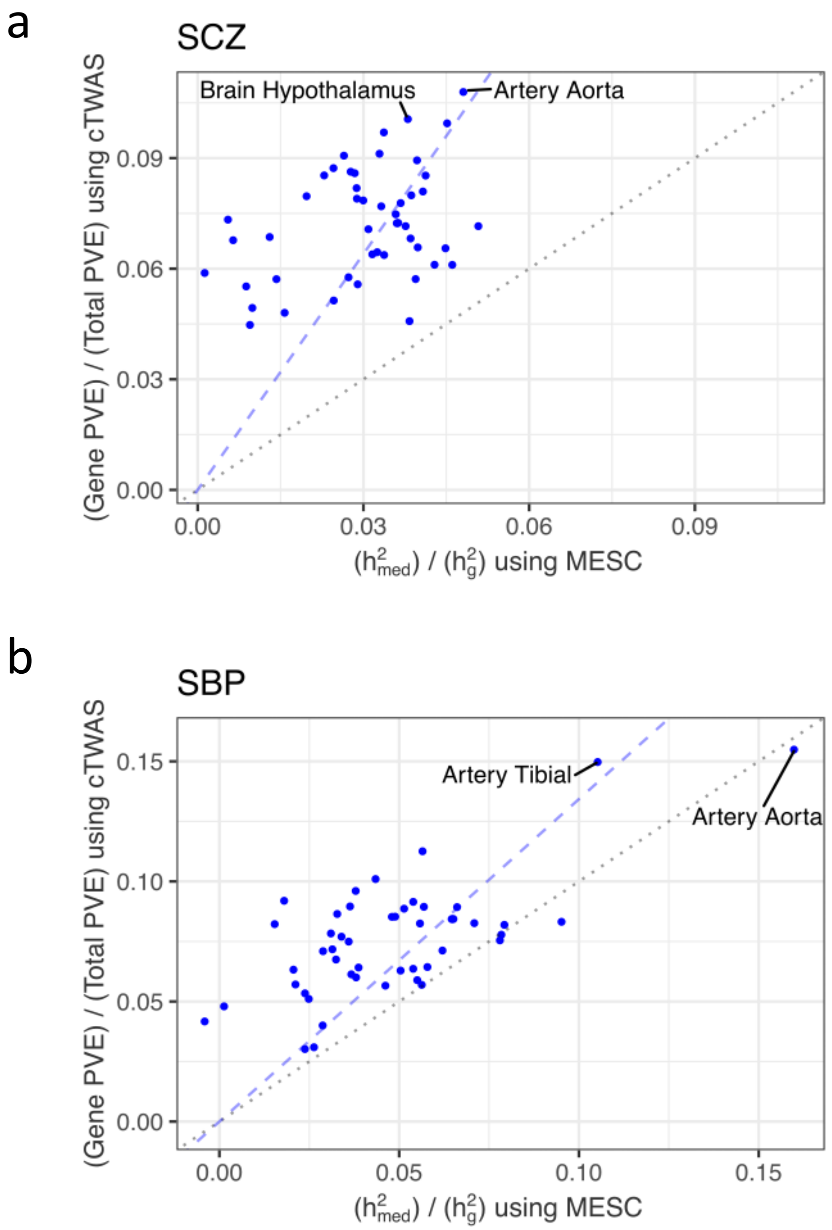

Supplementary Figure 13. Estimated proportions of mediated heritability by eQTLs for SCZ (**a**) and SBP (**b**), as estimated by MESC (X-axis) and by cTWAS (Y-axis). Each dot represents the result from one GTEx tissue. The black dotted lines denote equivalence between the methods, and the blue dashed lines denote the slope relating cTWAS and MESC mediated heritability estimates. Top two tissues by cTWAS were highlighted.

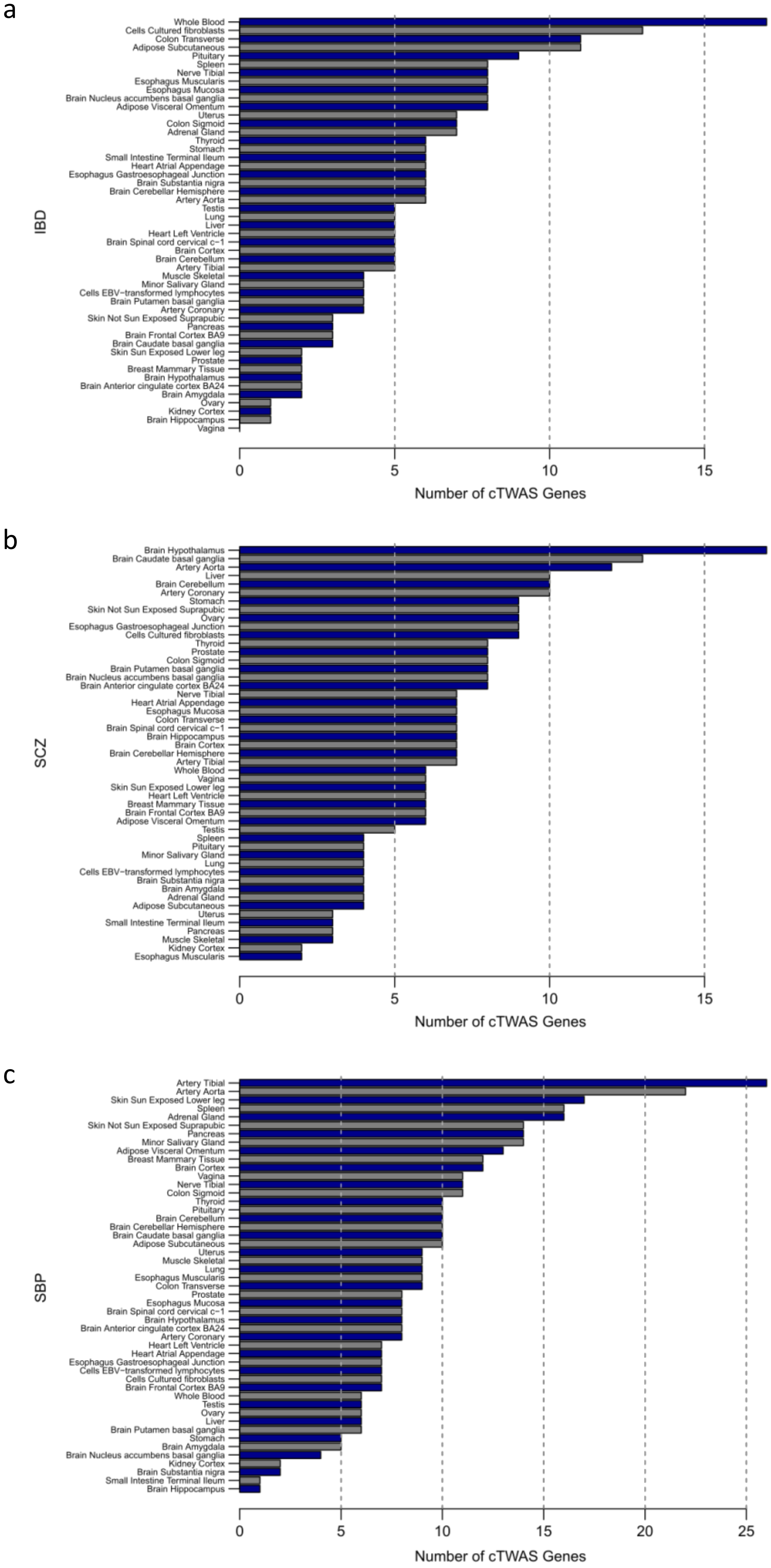

Supplementary Figure 14. Number of genes detected by cTWAS, at PIP > 0.8, across all tissues, for IBD (**a**), SCZ (**b**), and SBP (**c**). Each bar represents the result from one GTEx tissue.

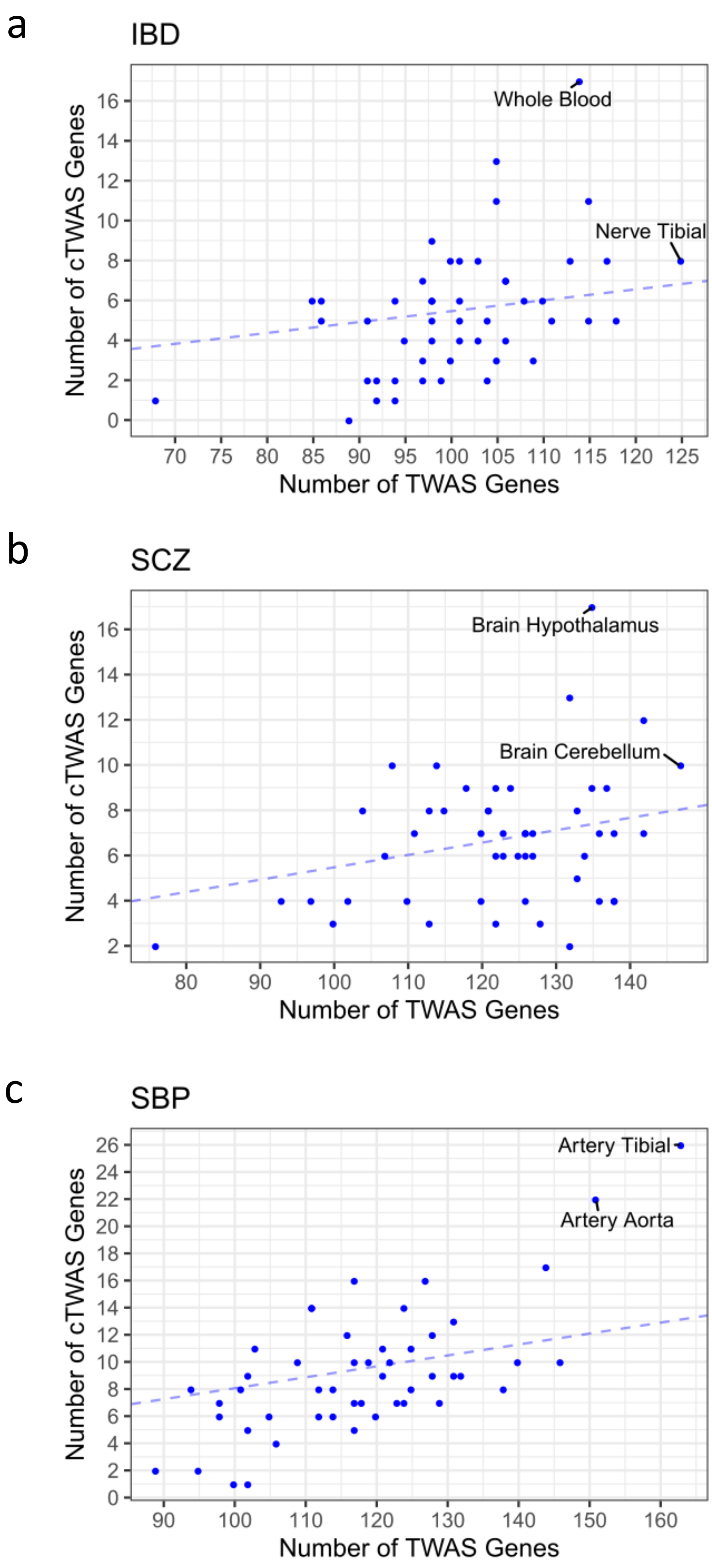

Supplementary Figure 15. Comparison of number of genes detected by TWAS and cTWAS across all tissues for IBD (a), SCZ (b), and SBP (c). cTWAS uses the cutoff of PIP > 0.8 (Y-axis), and TWAS uses a Bonferonni threshold (X-axis). Each dot represents the result from one GTEx tissue. The blue dashed lines denote the slopes relating the number of genes detected by cTWAS and TWAS.

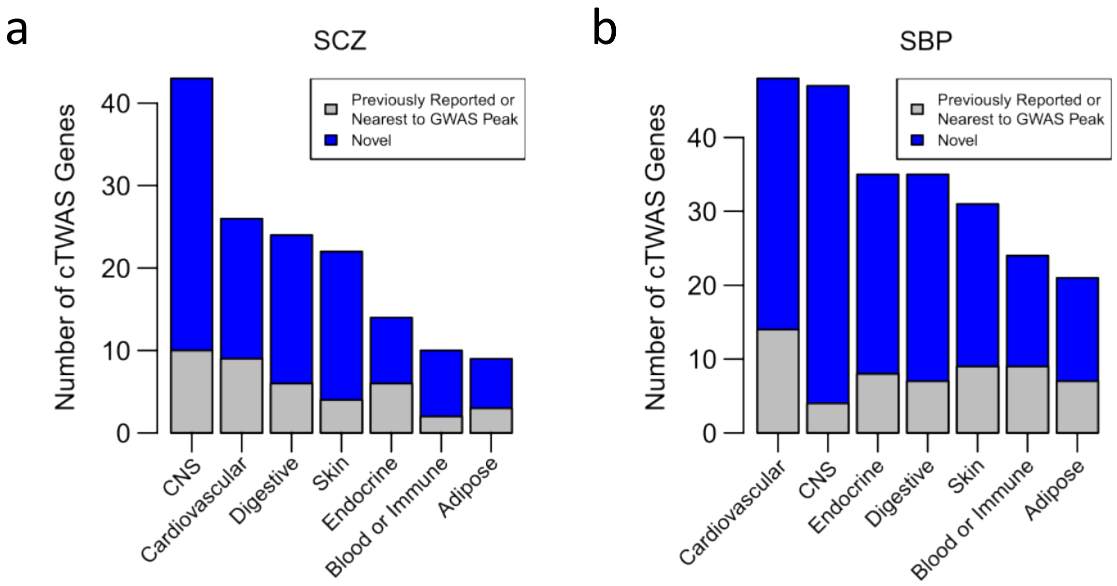

Supplementary Figure 16. Number of genes detected by cTWAS, at PIP > 0.8, across tissue groups for SCZ (a) and SBP (b). A gene was detected in a tissue group if it was detected in any of the tissues in that group. A gene was considered novel if it was not a previously reported candidate gene or if it was not the nearest gene to a genome-wide significant locus ("Nearest").

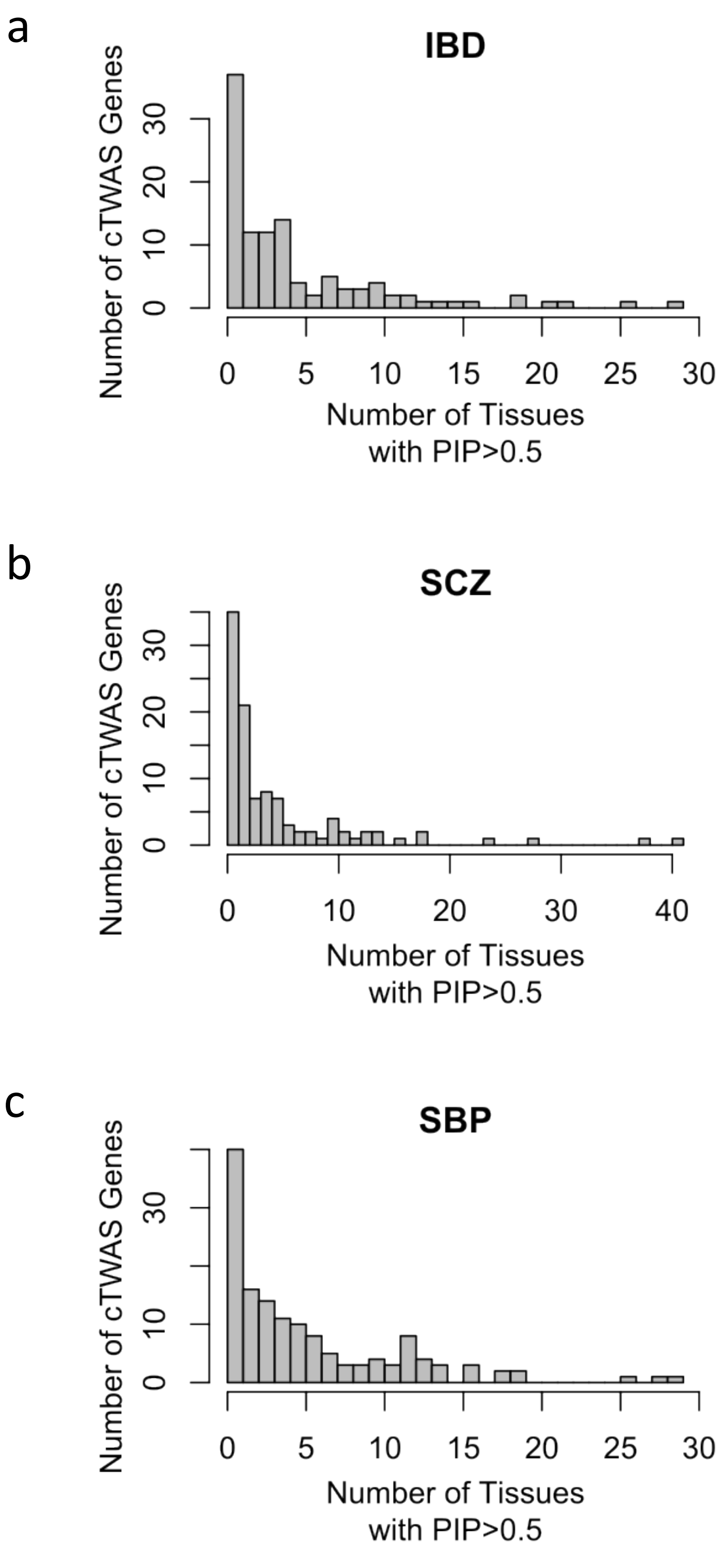

Supplementary Figure 17. Tissue specificity of genes detected by cTWAS. For all genes detected at PIP > 0.8 in at least one GTEx tissue, the number of tissues where the genes were detected at a relaxed threshold of PIP > 0.5 were shown. (a) 110 genes for IBD. (b) 104 genes for SCZ. (c) 142 genes for SBP.

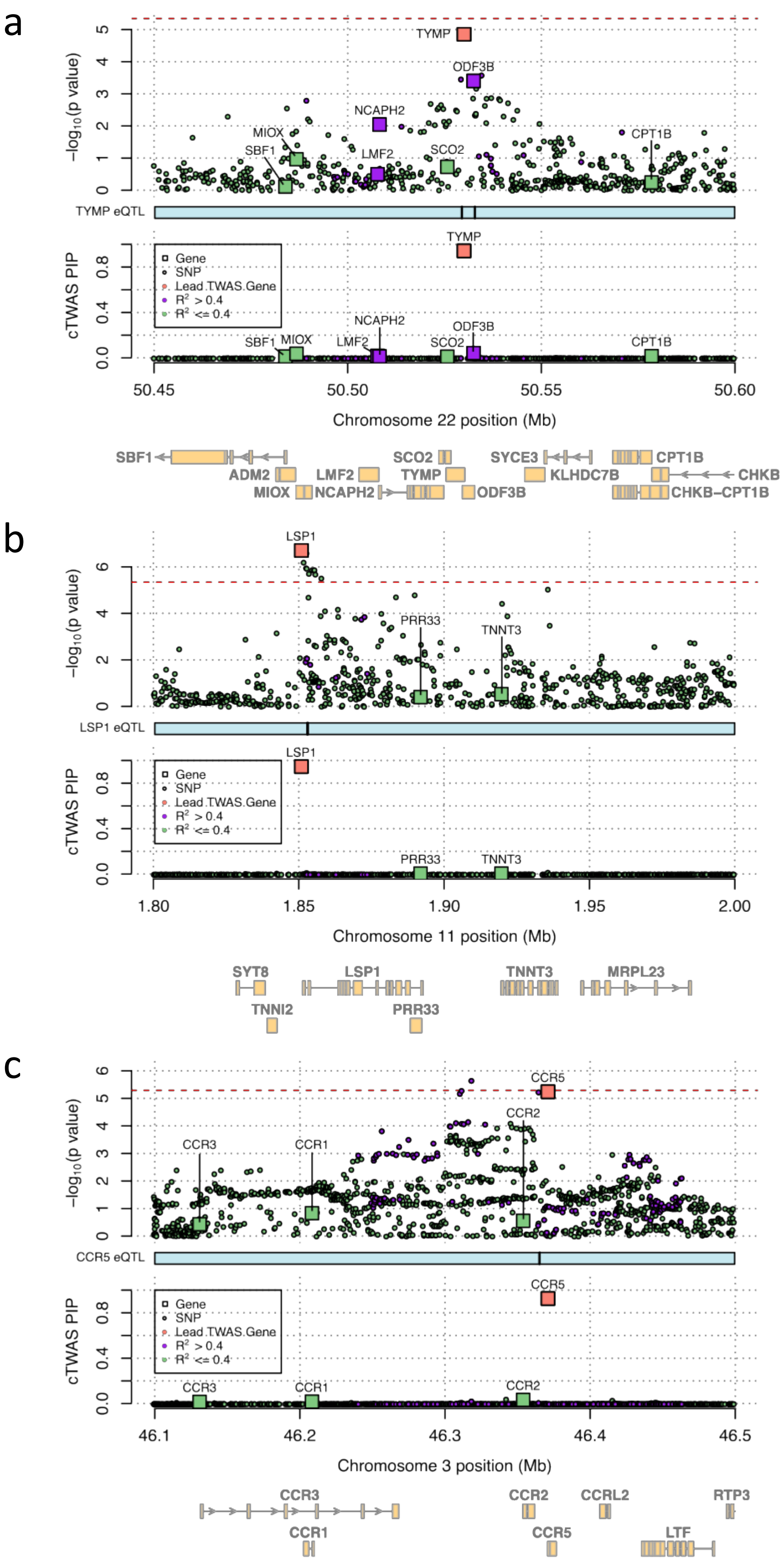

Supplementary Figure 18. cTWAS results at selected loci for IBD. a. The *TYMP* locus using the "Esophagus Mucosa" eQTL data. The figure legend is the same as in the locus plots of the main figures (e.g. see Fig. 4B). **b.** The *LSP1* locus using the "Esophagus Muscularis" eQTL data. **c.** The *CCR5* locus using the "White Blood" eQTL data. Exact p-values and PIPs are available in the Supplementary Data.

a

b

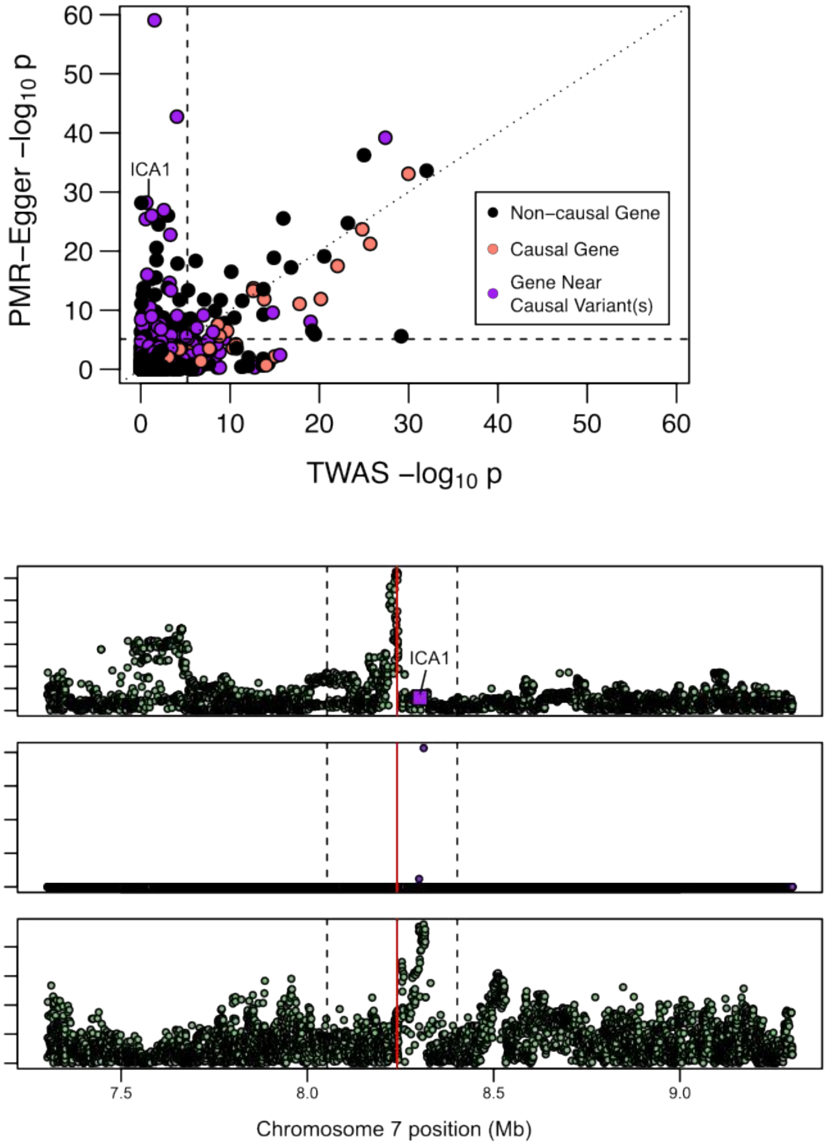

Supplementary Figure 19. Investigating false positives using PMR-Egger. Detailed results for a single simulation from the high PVE scenario. **a.** Significance by TWAS and PMR-Egger for 6,454 genes analyzed by both methods. Vertical and horizontal dashed lines denote Bonferroni significance thresholds for TWAS and PMR-Egger, respectively. Diagonal dotted line denotes equivalence between the two methods. "Causal genes" have a true effect on the simulated trait. "Genes near causal variant(s)" are non-causal genes within 100kb of variants with true effect on the simulated trait. "Non-causal genes" are other genes not in these categories, including those potentially confounded by genes. **b.** GWAS and eQTL summary statistics for variants at the *ICA1* locus. The top panel shows the magnitude of the GWAS z-scores. The purple square is the TWAS z-score of the *ICA1* gene. The middle panel shows the magnitude of the weights from the FUSION lasso-based eQTL prediction model of *ICA1*. The bottom panel shows the magnitude of the eQTL z-scores for ICA1. In all panels, the solid red lines denote the location of a simulated causal variant. The black dashed lines denote the boundaries for PMR-Egger input (+/-100kb from gene body).

Supplemental Table Legends

Supplementary Table 1. cTWAS results of all analyzed genes for LDL using liver eQTL data. The "id" and "genename" columns are Ensembl gene IDs and gene symbols. The "chrom" and "pos" columns are the genomic positions of the first eQTL in the prediction model for each gene. Note that eQTL positions rather than TSS positions are reported here because cTWAS uses eQTL positions to assign genes to regions for analysis. The "region_tag" column is a unique identifier of the region that each gene was analyzed in. The "cs_index" column denotes the confidence set that each gene is assigned to within a region; values of 0 indicate genes that were either unassigned or assigned to an impure confidence set (see Methods). The "PIP", "tau2", and "PVE" columns are the posterior inclusion probabilities, effect sizes, and proportions of variance explained using cTWAS for each gene. The "z" and "num_eqtl" columns are the TWAS z-scores and the number of eQTL in the gene prediction models after harmonization for each gene.

Supplementary Table 2. cTWAS results of silver standard and bystander genes for LDL using liver eQTL data. The "annotation" column denotes whether each gene is in the silver standard ("known") or bystander ("bystander") gene lists. Column legends for other columns are the same as in Supplementary Table 1. Silver standard genes without imputed expression are only listed with gene symbols and their number of eQTL (0). All other columns are shown as "NA"s. The "z"-score column shows the results from standard TWAS analysis with a genome-wide, Bonferroni-corrected significance threshold of $|z| > 4.56$.

Supplementary Table 3. GO enrichment analysis of the genes detected by cTWAS at PIP > 0.8 for LDL cholesterol using liver eQTL data. The "Term" and "DB" columns are the GO terms and database names. The "Overlap" column is the number of detected genes divided by the total number of genes in each term. The "P.value" and "Adjusted.P.value" columns are the p-values for enrichment of detected genes in each term using the Fisher exact test before and after multiple testing correction using B-H. The "Odds.Ratio" column is the ratio of the odds of detected genes being in a term relative to the odds of undetected genes being in a term. The "Combined.Score" column is a metric reported by Enrichr for ranking enriched terms; it is the product of the log p-value and the z-score of deviation from expected rank for each term. The "Genes" column lists the detected genes under each term.

Supplementary Table 4. GO enrichment analysis of 69 silver standard genes for LDL cholesterol. Column legends are the same as in Supplementary Table 2.

Supplementary Table 5. GO enrichment analysis using MAGMA for LDL cholesterol. The "Category" and "GeneSet" columns are the database names and GO terms. The "N_genes" and "N_overlap" columns are the total number of genes and the number of genes identified from the GWAS by MAGMA in each term. The "p" and "adjP" columns are the p-values for enrichment of genes identified from the GWAS in each term before and after multiple testing correction using B-H. The "genes" column lists the genes identified from the GWAS by MAGMA in each term.

Supplementary Table 6. Estimated parameters and proportion of mediated heritability using cTWAS across all tissues for IBD, SCZ, and SBP. The "trait" column is the abbreviated trait name for each analysis. The "tissue" column is the tissue used in each analysis. The "prior_g" and "prior_v" columns are the estimated prior inclusion probabilities for genes and variants in each analysis. The "prior_var_g" and "prior_var_v" columns are the estimated prior effect sizes for genes and variants in each analysis. The "enrich" column is the ratio of "prior_g" to "prior_v" and is a measure of the importance of gene inclusion relative to variant inclusion. The "pve_g" and "pve_v" columns are the proportions of trait variance attributable to genes and variants in each analysis. The "h2" column is the total heritability from all genetic variants and genes, computed as the sum of "pve_g" and "pve_v". The "prop_h2_g" column is the proportion of total heritability mediated by genes, computed as "pve_g" / "h2".

Supplementary Table 7. Proportion of mediated heritability using MESC across all tissues for IBD, SCZ, and SBP. The "trait" column is the abbreviated trait name for each analysis. The "weight" column is the tissue used in each analysis. The "pve_g" and "pve_v" columns are the proportions of trait variance attributable to genes and variants in each analysis. The "h2" column is the total heritability, computed as the sum of "h2med" and "h2nonmed". The "prop_h2_g" column is the proportion of total heritability mediated by genes, computed as "h2med" / "h2". Note that expression scores for "Kidney_Cortex" tissue were not available from MESC.

Supplementary Table 8. IBD genes detected by cTWAS at PIP > 0.8 in the Blood/Immune and Digestive tissue groups. The "genename" and "ensembl_gene_id" columns are gene symbols and Ensembl gene IDs. The "chromosome" and "start_position" columns are the genomic positions of the transcription start site for each gene. The "max_pip tissue" and "max_pip" columns denote the tissue with the highest PIP for each gene and its corresponding PIP. The "region_tag_tissue" column is a unique identifier of the region that each gene was analyzed in, for the tissue with the maximum PIP. The "z_tissue" and "num_eqtl_tissue" columns are the TWAS z-scores and the number of eQTL in the gene prediction models after harmonization for each gene, for the tissue with the maximum PIP. The "nearest_region_peak" column indicates if each gene is the nearest gene to the maximum GWAS signal in the region. The "distance_region_peak" column is the number of bases to the maximum GWAS signal in the region of each gene. The "which_nearest_region_peak" column lists the gene(s) nearest the maximum GWAS signal in the region of each gene; "-" denotes an unnamed gene. The "nearby" column indicates if each gene is within 500kb of a genome-wide significant locus. The "nearest" column indicates if each gene is the nearest gene to a genome-wide significant locus. The "distance" column is the number of bases to the nearest genome-wide significant locus, for genes that are "nearby" a genome-wide significant locus. The "which_nearest" column lists the gene nearest to a genome-wide significant locus, for genes that are "nearby" a genome-wide significant locus. The "known" column indicates genes that are on the silver standard gene list for IBD. The "GO_cTWAS" column lists the GO terms significantly enriched for cTWAS genes (Supplementary Table 10) that are associated with each gene. The "GO_silver" column lists the GO terms significantly enriched for silver standard genes (Supplementary Table 11) that are associated with each gene. The "GO_MAGMA" column lists the GO terms significantly enriched using MAGMA (Supplementary Table 12) that are associated with each gene. For all three GO annotations, a maximum of 5 significant terms per gene are shown, ordered by odds ratios (cTWAS, silver standard) or p-values (MAGMA).

Supplementary Table 9. GO enrichment analysis using WebGestalt of genes detected by cTWAS at PIP > 0.8 for IBD using eQTLs in the Blood/Immune or Digestive tissue groups. The "geneSet" and "description" columns are the GO terms and names. The "size" column is the total number of genes in each term. The "overlap" column is the number of detected genes in each term. The "expect" column is the expected number of detected genes in each term. The "enrichment" column is the ratio of the "overlap" and "expect" columns. The "pValue" and "FDR" columns are the p-values for enrichment of detected genes in each term before and after correcting for multiple comparisons. The "Genes" column lists the detected genes in each term.

Supplementary Table 10. GO enrichment analysis using Enrichr of genes detected by cTWAS at PIP > 0.8 for IBD using eQTLs in the Blood/Immune or Digestive tissue groups. Column legends are the same as in Supplementary Table 3.

Supplementary Table 11. GO enrichment analysis of silver standard genes for IBD. Column legends are the same as in Supplementary Table 3.

Supplementary Table 12. GO enrichment analysis using MAGMA for IBD. Column legends are the same as in Supplementary Table 5.

Supplementary Data Legend.
cTWAS results of all analyzed genes across all tissues for IBD, SCZ, and SBP. Results are stored separately for each combination of traits and tissues. Column legends for each set of results are the same as in Supplementary Table 1.
