# Supplementary material for: Adjusting for genetic confounders in transcriptome-wide association studies improves discovery of risk genes of complex traits
Source: Nat Genet. 2024 Jan 26;56(2):336–47. doi: 10.1038/s41588-023-01648-9 (PMC10864181; doi:10.1038/s41588-023-01648-9)
Supplement: Supplementary file 2 — Reporting Summary [file 41588_2023_1648_MOESM2_ESM.pdf]

nature portfolio

Corresponding author(s): Xin He, Matthew Stephens, Siming Zhao

Last updated by author(s): 2023-7-15

Reporting Summary

Nature Portfolio wishes to improve the reproducibility of the work that we publish. This form provides structure for consistency and transparency in reporting. For further information on Nature Portfolio policies, see our Editorial Policies and the Editorial Policy Checklist.

Statistics

For all statistical analyses, confirm that the following items are present in the figure legend, table legend, main text, or Methods section.

n/a	Confirmed	
☐	☒	The exact sample size (n) for each experimental group/condition, given as a discrete number and unit of measurement
☐	☒	A statement on whether measurements were taken from distinct samples or whether the same sample was measured repeatedly
☐	☒	The statistical test(s) used AND whether they are one- or two-sided<br>*Only common tests should be described solely by name; describe more complex techniques in the Methods section.*
☐	☒	A description of all covariates tested
☐	☒	A description of any assumptions or corrections, such as tests of normality and adjustment for multiple comparisons
☐	☒	A full description of the statistical parameters including central tendency (e.g. means) or other basic estimates (e.g. regression coefficient) AND variation (e.g. standard deviation) or associated estimates of uncertainty (e.g. confidence intervals)
☐	☒	For null hypothesis testing, the test statistic (e.g. F, t, r) with confidence intervals, effect sizes, degrees of freedom and P value noted<br>*Give P values as exact values whenever suitable.*
☐	☒	For Bayesian analysis, information on the choice of priors and Markov chain Monte Carlo settings
☒	☐	For hierarchical and complex designs, identification of the appropriate level for tests and full reporting of outcomes
☐	☒	Estimates of effect sizes (e.g. Cohen's d, Pearson's r), indicating how they were calculated

Our web collection on statistics for biologists contains articles on many of the points above.

Software and code

Policy information about availability of computer code

Data collection	Data collection is not part of this study
Data analysis	All analysis were performed on the high throughput research computing clusters (midway and CRI) at University of Chicago. We used R 3.6.1 for statistical analysis. cTWAS v0.1.29 (https://github.com/xinhe-lab/ctwas) were used for results presented. The method section contains details of comparator software we used. Comparator software versions: MR-JTI: DOI 10.5281/zenodo.4164740. FUSION and coloc (http://gusevlab.org/projects/fusion/, accessed Feb, 2021), FOCUS, version 0.6. MR-locus, mrlocus_0.0.25. PMR, v1.0. SMR, v1.03.

For manuscripts utilizing custom algorithms or software that are central to the research but not yet described in published literature, software must be made available to editors and reviewers. We strongly encourage code deposition in a community repository (e.g. GitHub). See the Nature Portfolio guidelines for submitting code & software for further information.

Data

Policy information about availability of data

All manuscripts must include a data availability statement. This statement should provide the following information, where applicable:

- Accession codes, unique identifiers, or web links for publicly available datasets
- A description of any restrictions on data availability
- For clinical datasets or third party data, please ensure that the statement adheres to our policy

Genotype data from UK Biobank are available through the UK Biobank data access process (see http://www.ukbiobank.ac.uk/register-apply/). GTEx v7 Adipose

tissue dataset gene prediction models,http://gusevlab.org/projects/fusion/.Publically available summary statistics for LDL, SBP, and IBD were obtained from the IEU OpenGWAS project (https://gwas.mrcieu.ac.uk/) using GWAS IDs “ukb-d-30780_irnt” (LDL), “ukb-a-360” (SBP), and “ebi-a-GCST004131” (IBD). Publically available summary statistics for SCZ from the Psychiatric Genetics Consortium and the CardiffCOGS study were obtained from the authors via the link provided in the manuscript (https://doi.org/10.1038/s41588-018-0059-2): http://walters.psycm.cf.ac.uk/ Publicly available prediction models for 49 GTEx tissues from PredictDB: https://predictdb.org/post/2021/07/21/gtex-v8-models-on-eqtl-and-sqtl/.

Human research participants

Policy information about studies involving human research participants and Sex and Gender in Research.

Reporting on sex and gender	The study used publicly available GWAS summary statistics.
Population characteristics	NA
Recruitment	NA
Ethics oversight	NA

Note that full information on the approval of the study protocol must also be provided in the manuscript.

Field-specific reporting

Please select the one below that is the best fit for your research. If you are not sure, read the appropriate sections before making your selection.

☒ Life sciences ☐ Behavioural & social sciences ☐ Ecological, evolutionary & environmental sciences

For a reference copy of the document with all sections, see nature.com/documents/nr-reporting-summary-flat.pdf

Life sciences study design

All studies must disclose on these points even when the disclosure is negative.

Sample size	Sample size is provided with each analysis performed in the manuscript. We chose a few sample sizes for simulation studies to match current typical GWAS cohort sizes. For real data application, we used published GWAS summary statistics.
Data exclusions	In simulations, we filtered samples to only keep "White British", removed samples with missing information, mismatches between self-reported and genetic sex, or "outliers" as defined by UK Biobank. We also removed any individuals that have close relatives in the cohort.
Replication	All results are reproducible.
Randomization	In simulation studies, we used genotype data from UK biobank by randomly selecting 80,000 samples. We then filtered samples to only keep "White British", removed samples with missing information, mismatches between self-reported and genetic sex, or "outliers" as defined by UK Biobank. We also removed any individuals that have close relatives in the cohort. This ended up with a cohort of n = 45,087 samples. In real data application, To ease computation, we randomly selected 10% of these samples to serve as the LD reference panel.
Blinding	The individuals are blind to investigators.

Reporting for specific materials, systems and methods

We require information from authors about some types of materials, experimental systems and methods used in many studies. Here, indicate whether each material, system or method listed is relevant to your study. If you are not sure if a list item applies to your research, read the appropriate section before selecting a response.

Materials & experimental systems

n/a	Involved in the study
☒	☐ Antibodies
☒	☐ Eukaryotic cell lines
☒	☐ Palaeontology and archaeology
☒	☐ Animals and other organisms
☒	☐ Clinical data
☒	☐ Dual use research of concern

Methods

n/a	Involved in the study
☒	☐ ChIP-seq
☒	☐ Flow cytometry
☒	☐ MRI-based neuroimaging
